# Vpu Binds Directly to Tetherin and Displaces It from Nascent Virions

**DOI:** 10.1371/journal.ppat.1003299

**Published:** 2013-04-25

**Authors:** Matthew W. McNatt, Trinity Zang, Paul D. Bieniasz

**Affiliations:** Howard Hughes Medical Institute, Aaron Diamond AIDS Research Center and Laboratory of Retrovirology, The Rockefeller University, New York, New York, United States of America; Harvard Medical School, United States of America

## Abstract

Tetherin (Bst2/CD317/HM1.24) is an interferon-induced antiviral host protein that inhibits the release of many enveloped viruses by tethering virions to the cell surface. The HIV-1 accessory protein, Vpu, antagonizes Tetherin through a variety of proposed mechanisms, including surface downregulation and degradation. Previous studies have demonstrated that mutation of the transmembrane domains (TMD) of both Vpu and Tetherin affect antagonism, but it is not known whether Vpu and Tetherin bind directly to each other. Here, we use cysteine-scanning mutagenesis coupled with oxidation-induced cross-linking to demonstrate that Vpu and Tetherin TMDs bind directly to each other in the membranes of living cells and to map TMD residues that contact each other. We also reveal a property of Vpu, namely the ability to displace Tetherin from sites of viral assembly, which enables Vpu to exhibit residual Tetherin antagonist activity in the absence of surface downregulation or degradation. Elements in the cytoplasmic tail domain (CTD) of Vpu mediate this displacement activity, as shown by experiments in which Vpu CTD fragments were directly attached to Tetherin in the absence of the TMD. In particular, the C-terminal α-helix (H2) of Vpu CTD is sufficient to remove Tetherin from sites of viral assembly and is necessary for full Tetherin antagonist activity. Overall, these data demonstrate that Vpu and Tetherin interact directly via their transmembrane domains enabling activities present in the CTD of Vpu to remove Tetherin from sites of viral assembly.

## Author Summary

At the cell surface, HIV-1 particles are assembled and then released to infect new cells. However, an anti-viral host restriction factor, Tetherin, can tether outgoing virions to the infected cell surface, preventing their dissemination. HIV-1 overcomes this block through the expression of the viral accessory protein Vpu, which antagonizes Tetherin. In this study, we demonstrate that the domains of Vpu and Tetherin that are embedded in the outer cell membrane bind directly to each other within the membrane, and we identify amino acids that participate directly in the interaction between these two proteins. After binding to Tetherin, Vpu can induce its removal from the cell surface and degradation. However, a mutant Vpu lacking these activities retains some capacity to antagonize Tetherin. We show that this residual activity requires a particular portion of the intracellular domain of Vpu and is manifested as an ability to displace Tetherin from sites of viral assembly, without affecting the overall level of Tetherin at the cell surface. These data indicate that Vpu directly binds to Tetherin and then employs multiple mechanisms, including displacement, to counteract Tetherin's ability to restrict virus particle release.

## Introduction

Tetherin is an antiviral protein that can inhibit the release of a broad-spectrum of enveloped viruses, including retroviruses [1]–[7], filoviruses [4], [8]–[10], arenaviruses [9], [10], rhabdoviruses [11] and herpesviruses [6], [12]. The Tetherin protein is a type-II single-pass transmembrane domain (TMD) protein that is also appended with a C-terminal glycophosphatidylinositol (GPI) moiety as a second membrane anchor [13]. Between the membrane anchors is a coiled-coil (CC) domain that is covalently linked to a second Tetherin molecule by three intermolecular disulfide bonds [14]–[18]. Both membrane anchors and the CC domain are necessary for activity, and the membrane anchors drive incorporation of Tetherin dimers into virion envelopes [14], [19]. The primary sequence of the Tetherin protein is relatively unimportant for activity, leading to a model in which Tetherin directly tethers virions to infected cells simply through the partition of its membrane anchors into both virion and cell membranes [14]. Consistent with this notion, Tetherin colocalizes with virions at the cell surface and can be observed to reside between cell and tethered virion membranes by electron microscopy [14], [19], [20].

Human immunodeficiency virus type-1 (HIV-1) can overcome the restriction imposed by Tetherin through the activity of viral protein U (Vpu) [1], [2]. Vpu is a single-pass type-I membrane protein containing an amino-terminal TMD and a carboxy-terminal cytoplasmic tail domain (CTD). The Vpu CTD consists of two α-helices (H1 and H2), linked by a short loop [21], [22]. The loop contains two phosphorylatable serine residues, S52 and S56 [23], [24], that are required to recruit β-TrCP and its associated Skp1/Cullin/F-Box (SCF) ubiquitin ligase [25]. Vpu can mediate the surface downregulation [2], [26]–[32] and degradation [33]–[36] of Tetherin, and the phosphorylatable serine residues that are necessary for β-TrCP recruitment are also required for the surface downregulation [2], [28]–[31], [33]–[36] and degradation [28], [31], [33]–[36] of Tetherin.

Analysis of sequences from various mammalian species has revealed that Tetherin has evolved under positive or diversifying selection [37], [38] and that, consequently, the ability of Vpu to antagonize Tetherin is host-species specific [37]–[40]. Experiments in which domains of human Tetherin were exchanged with those of Tetherin proteins from other primates, revealed that the TMD of Tetherin governs Vpu sensitivity [37]–[40]. Indeed, mutations at two positions in the TMD of human Tetherin, which mimic sequences found in rhesus macaques and African green monkeys, conferred resistance to Vpu antagonism [38]. A few studies have shown that Vpu and Tetherin can be coimmunoprecipitated in a manner that is dependent on Tetherin and Vpu TMDs sequences [34], [40]–[44]. These studies suggest, but do not prove, that Vpu and Tetherin may interact via their TMDs. In this study, we use cysteine-scanning mutagenesis combined with oxidative cross-linking [45] to: (i) demonstrate conclusively that Vpu and Tetherin indeed bind directly to each other in living cell membranes via sequences in their respective TMDs and (ii) identify TMD amino acids that are in close proximity to each other in Vpu-Tetherin complexes.

While the binding of Vpu to Tetherin appears to be necessary for antagonism, it is clearly not sufficient. Indeed, several studies have reported that Vpu antagonizes Tetherin by causing CTD-dependent sequestration into internal compartments [26]–[32] and degradation via β-TrCP induced ubiquitination [31], [33]–[36], [46]. Nonetheless, Vpu apparently induces Tetherin downregulation and degradation in only a subset of cell types, even though all cells tested support Vpu activity [28], [47], [48]. Additionally, a Vpu (Vpu^2/6^) mutant, in which the two phosphorylated serine residues are mutated, and therefore cannot recruit β-TrCP [25], retains some ability to antagonize Tetherin without inducing cell surface downregulation or degradation [28], [33], [34], [49]. These data suggest that Vpu can inhibit Tetherin function independently of downregulation and degradation. Here, we demonstrate that this is indeed the case, and we show that the Vpu CTD has an autonomous activity, conferred largely by the H2 domain that causes displacement of the Tetherin protein from assembling virions. Moreover, we find that an ExxxLV motif within H2 that has previously been reported to be required for downregulation and degradation of Tetherin [50] is also required for the efficient displacement of Tetherin from sites of virion assembly at the cell surface.

## Results

### Tetherin and Vpu directly interact through their TMDs

**Figure 1:**
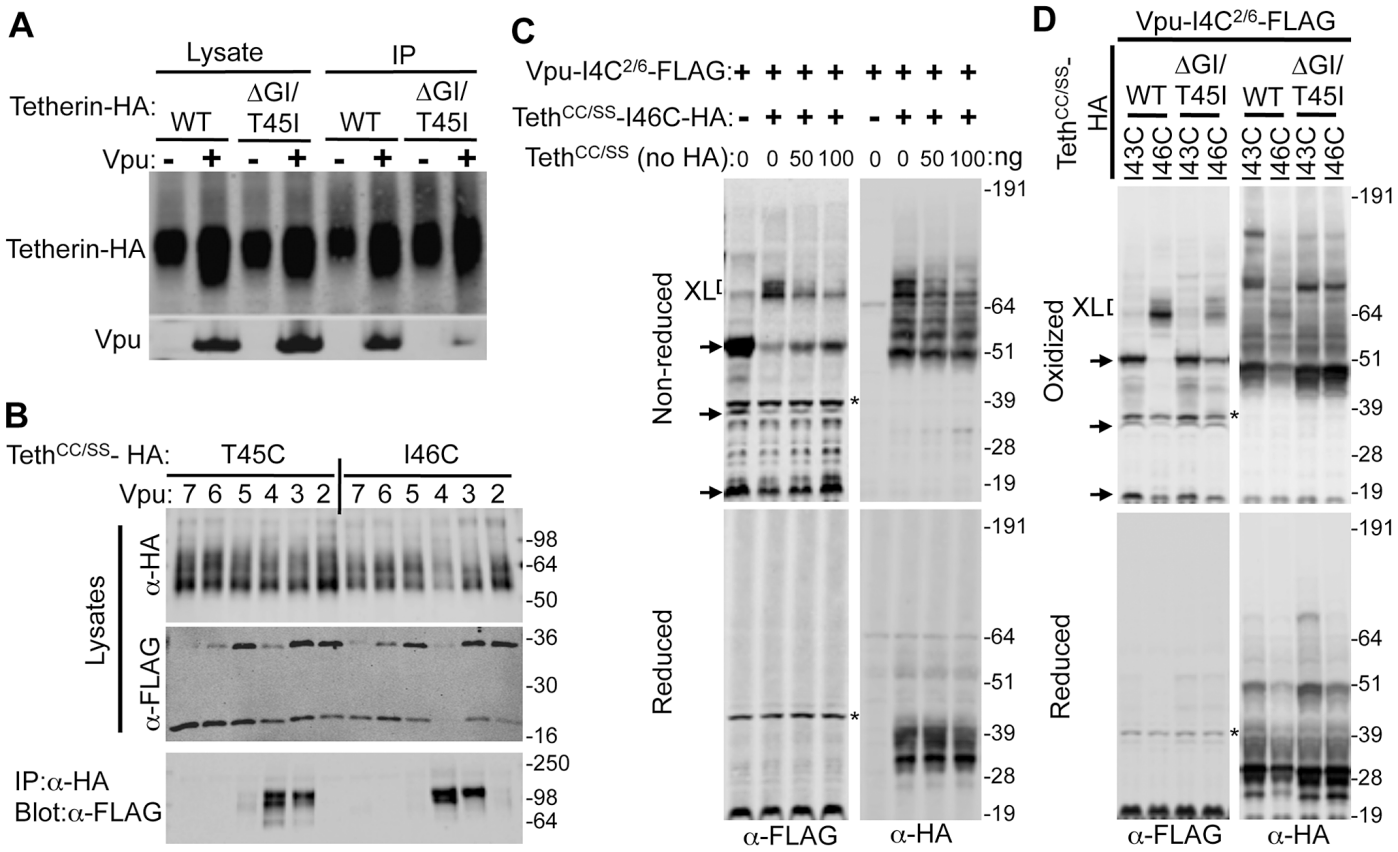
Tetherin and Vpu directly interact via their TMDs. (**A**) 293T cells stably expressing Tetherin-HA or TetherinΔGI-T45I-HA were transfected with a plasmid expressing Vpu^2/6^-FLAG or an empty vector and subjected to immunoprecipitation with an α-HA antibody. Unfractionated cell lysates and immunoprecipitates were subjected to western blot analysis with the indicated antibodies. (**B**) 293T cells were co-transfected with plasmids encoding Tetherin^CC/SS^-T45C-HA or Tetherin^CC/SS^-I46C-HA along with plasmids expressing individual Vpu^2/6^-FLAG transmembrane proteins, mutated to encode cysteine at amino acid positions 2, 3, 4, 5, 6, or 7, as indicated). Cells were disrupted by sonicating in the presence of oxidizer and protein denatured using SDS, and immunoprecipitation performed using α-HA antibodies. Unfractionated cell lysates and immunoprecipitates were subjected to Western blot analysis with the indicated antibodies. (**C**) Whole cell lysates of 293T cells were co-transfected with a fixed amount of plasmids expressing Vpu-I4C^2/6^-FLAG and Tetherin^CC/SS^-I46C-HA, along with either an empty vector or increasing amounts (0, 50 or 100 ng) of plasmid expressing untagged Tetherin^CC/SS^. Cell lysates were divided into two and were either untreated (non reduced) or treated with reducing agent (reduced) and then subjected to Western blot analysis with the indicated antibodies. ‘XL’ and ‘arrows’ indicate Vpu-Tetherin or Vpu-Vpu cross-linked species, respectively. (**D**) 293T cells were co-transfected with plasmids encoding Vpu-I4C^2/6^-FLAG and plasmids expressing either WT or ΔGI-T45I mutant Tetherin^CC/SS^-HA WT or ΔGI-T45I TMD that also included I43C or I46C mutations. Samples were sonicated in the presence of oxidizer and then divided into two. Thereafter, samples were not subjected to further treatment (oxidized, upper panels) or were treated with reducing agent, (reduced, lower panels). Samples were then subjected to Western blot analysis with the indicated antibodies. For (**B**–**D**) the migration of markers of the indicated molecular weight (kDa) is indicated, and asterisks indicate nonspecific bands.

We and others have demonstrated that the HIV-1 Vpu protein selectively antagonizes Tetherin proteins from particular species, in part because Tetherin has evolved under positive selection [37], [38]. As part of this work, we derived the human TetherinΔGI-T45I mutant that is resistant to antagonism by HIV-1 Vpu due to mutations at two positions in the TMD (a T45I substitution and a G27/I28 deletion) [38]. Consistent with previous observations [34], [40]–[44], WT Tetherin-HA was efficiently coprecipitated with Vpu^2/6^-FLAG, whereas minimal amounts of TetherinΔGI-T45I-HA were coprecipitated (Figure 1A). These data suggest that Tetherin and Vpu might physically interact, and that their TMDs are determinants of such an interaction.

In order to determine whether Vpu and Tetherin bind directly to each other, in their natural environment (i.e. the membranes of living cells), we attempted to generate chemical crosslinks between the two proteins under native conditions. Specifically, panels of Vpu and Tetherin TMD mutants were generated in which each mutant protein harbored a single cysteine residue in its TMD domain. If Tetherin and Vpu directly bind to each other, a disulfide bond should form between proximal cysteines if the two proteins are coexpressed in an oxidizing environment [45]. To simplify this analysis, we used Tetherin proteins in which the two naturally occurring cysteines in the cytoplasmic domain were mutated to serine (Tetherin^CC/SS^-HA). This did not affect Tetherin activity or sensitivity to antagonism by Vpu (unpublished observations), but removed a potential source of confounding crosslinks. We also used a mutant Vpu^2/6^-FLAG protein, so that any complexes that might form would not be degraded. Initially, we attempted to generate cysteine cross-links at residues proximal to Tetherin T45 to assess whether this functionally important residue could directly contact Vpu. Thus, Tetherin-HA proteins bearing cysteine residues at positions T45 and I46 were coexpressed with Vpu^2/6^-FLAG proteins encoding cysteines at positions 2 through 7. After oxidation, cell lysis, denaturation and immunoprecipitation, proteins were separated on non-reducing SDS-PAGE gels and subjected to Western blot analyses. As expected, the bulk of the Tetherin-HA protein migrated as a homodimer of ∼60 kDa (Figure 1B) and the majority of the Vpu^2/6^-FLAG proteins migrated as ∼17 kDa monomers or ∼35 kDa dimers when whole cell lysates were analyzed. However, in some of the anti-HA immunoprecipitates, Vpu^2/6^-FLAG was observed to migrate as a shifted ∼90–100 kDa species, consistent with the notion that one or two Vpu^2/6^-FLAG proteins were crosslinked to a Tetherin dimer (Figure 1B, lower panel). We observed cross-linked Tetherin-Vpu species only when particular residues in the TMD of two proteins were mutated to a cysteine. Specifically, Tetherin proteins bearing Cys residues at TM positions 45 and 46 formed cross-linked species with Vpu proteins modified with cysteines at positions 3 and 4, but not at positions 2, 5, 6 or 7 (Figure 1B).

To further confirm that the high molecular weight species were specifically crosslinked complexes that occurred as a result of Tetherin-Vpu binding, we performed a competition assay in which increasing levels of un-tagged and non-cross-linkable Tetherin^CC/SS^ were coexpressed with constant amounts of cross-linkable Tetherin^CC/SS^-(I46C)-HA and Vpu(I4C)-FLAG (Figure 1C). In this and subsequent experiments, whole cell lysates rather than immunoprecipitates were analyzed by Western blotting to determine whether cross-linked Vpu-Tetherin species were generated, which could be observed by the formation of a higher molecular weight FLAG-tagged protein species. Importantly, co-expression of the non-cross-linkable Tetherin reduced the levels of cross-linked Vpu-Tetherin-HA high molecular weight complexes migrating at ∼70 kDa (Figure 1C, upper panels “XL” label). Aliquots from these samples were reduced to dissociate the disulfide linked complexes and re-analyzed by Western blot to confirm that approximately equal levels of the tagged Tetherin and Vpu proteins were present under each condition (Figure 1C, lower panels). Note that the gel shown in Figure 1C and subsequent experiments were run using a different buffer system (NuPage, Invitrogen) that is less denaturing than that shown in Figure 1B (Bio-Rad) and, therefore, multiple higher-order molecular weight forms of Vpu (monomers, dimers, and trimers) can be observed under nonreducing conditions and also note that the crosslinked species migrates at an apparently lower molecular weight (Figure 1C, upper panel's arrows and XL label, respectively).

As a further test of the specificity of the crosslinking assay, a cross-linkable form of the TetherinΔGI-T45I-HA mutant was generated and co-expressed with Vpu-(I4C)-FLAG. This Vpu-resistant Tetherin mutant was also modified to contain a cysteine at position 46 (I46C) or at position 43 (I43C). As before, Tetherin^CC/SS^-(I46C)-HA was robustly cross-linked with Vpu-(I4C)-FLAG, yielding a protein species migrating at ∼70 kDa that was detected using antibodies to FLAG or HA (Figure 1D, upper panels “XL” label). However the abundance of the Tetherin-Vpu crosslinked complex was greatly reduced when the Vpu-resistant Tetherin^CC/SS^-ΔGI-T45I-(I46C)-HA was used. Overall, these data demonstrate that Tetherin and Vpu directly bind to each other via their TMDs in cell membranes, and that this interaction is inhibited by mutations in the Tetherin TMD that confer resistance to Vpu antagonism.

### Cysteine scanning mutagenesis of Tetherin and Vpu TMDs reveals multiple points of direct interaction

**Figure 2:**
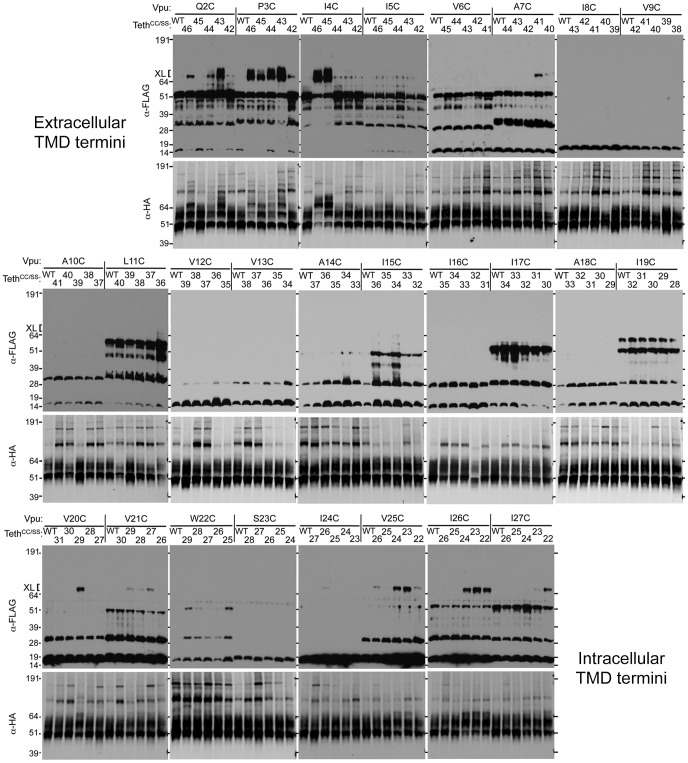
Mapping Vpu and Tetherin TMD residues that mediate interaction using a cysteine cross-linking assay. Each individual TMD cysteine-mutant Vpu protein was co-expressed with either Tetherin^CC/SS^-HA (“WT”), or with each of five individual TMD cysteine-mutant Tetherin^CC/SS^-HA proteins. The positions of the amino acids that were mutated to cysteine in the Vpu and Tetherin proteins are indicated above the blots. Transfected cells were treated with oxidizer and the cell lysates were analyzed using Western blots, probed with α-HA and α-FLAG antibodies. The migration of markers of the indicated molecular weights (kDa) is indicated, and bracket labeled “XL” indicate presumptive cross-linked Tetherin-Vpu products.

We applied the cysteine scanning mutagenesis and cross-linking technique to identify residues that participate in the interactions between Vpu and Tetherin. Each Vpu TMD cysteine mutant (residues 2 through 27) was co-expressed with a panel of five individual, consecutive Tetherin TMD cysteine mutants that were predicted to be in the vicinity of each introduced Vpu cysteine. As the Vpu mutation-scan progressed through the TMD, the register of the five Tetherin TMD cysteine mutants (between residues 22 and 46) was moved to match the Vpu progression. Overall, 130 individual Vpu-Tetherin cysteine-mutant pairs were tested for their ability to form disulfide-linked complexes. As a control, to reveal the background of higher molecular weight FLAG tagged Vpu-Vpu homo-oligomers, each cysteine-modified Vpu protein was also coexpressed with the non-cross-linkable Tetherin^CC/SS^-HA protein. Thus, Vpu-Tetherin cross-linked complexes were revealed by the presence of a new ∼70 kDa protein species on Vpu-FLAG blots, that was absent when the non-crosslinkable Tetherin^CC/SS^-HA was expressed (labeled “WT” with respect to the TMD) (Figure 2).

These experiments revealed four major points of proximity between Vpu and Tetherin TMDs (Figure 2). At the amino terminus of Vpu, (i.e. close to the outer leaflet of the cell membrane) VpuQ2C, P3C, and I4C formed cross-links with a few Tetherin cysteine residues. However, the most efficient cross-linking was observed between VpuI4C and TetherinI46C or T45C. Indeed, for these pairs of crosslinkable mutants, the majority of both the Vpu and the Tetherin proteins were present in a cross-linked complex (Figure 2, labeled as “XL”). Moving further into the TMD, VpuA7C cross-linked with moderate efficiency with TetherinL41C and at low efficiency with Tetherin P40C. At the carboxyl end of the Vpu TMD (near the cytoplasmic leaflet of the cell membrane), VpuV20C formed cross-links with TetherinL29C, and VpuV21C formed cross-links, albeit inefficiently, with TetherinL29C, I28C and G27C. At the extreme C-terminus of the Vpu TMD (and the N-terminus of the Tetherin TMD) cross-linking events were observed between VpuV25C and TetherinL24C/L23C/L22C, VpuI26C and TetherinL24C/L23C/L22C, as well as VpuI27C and TetherinL22C.

**Figure 3:**
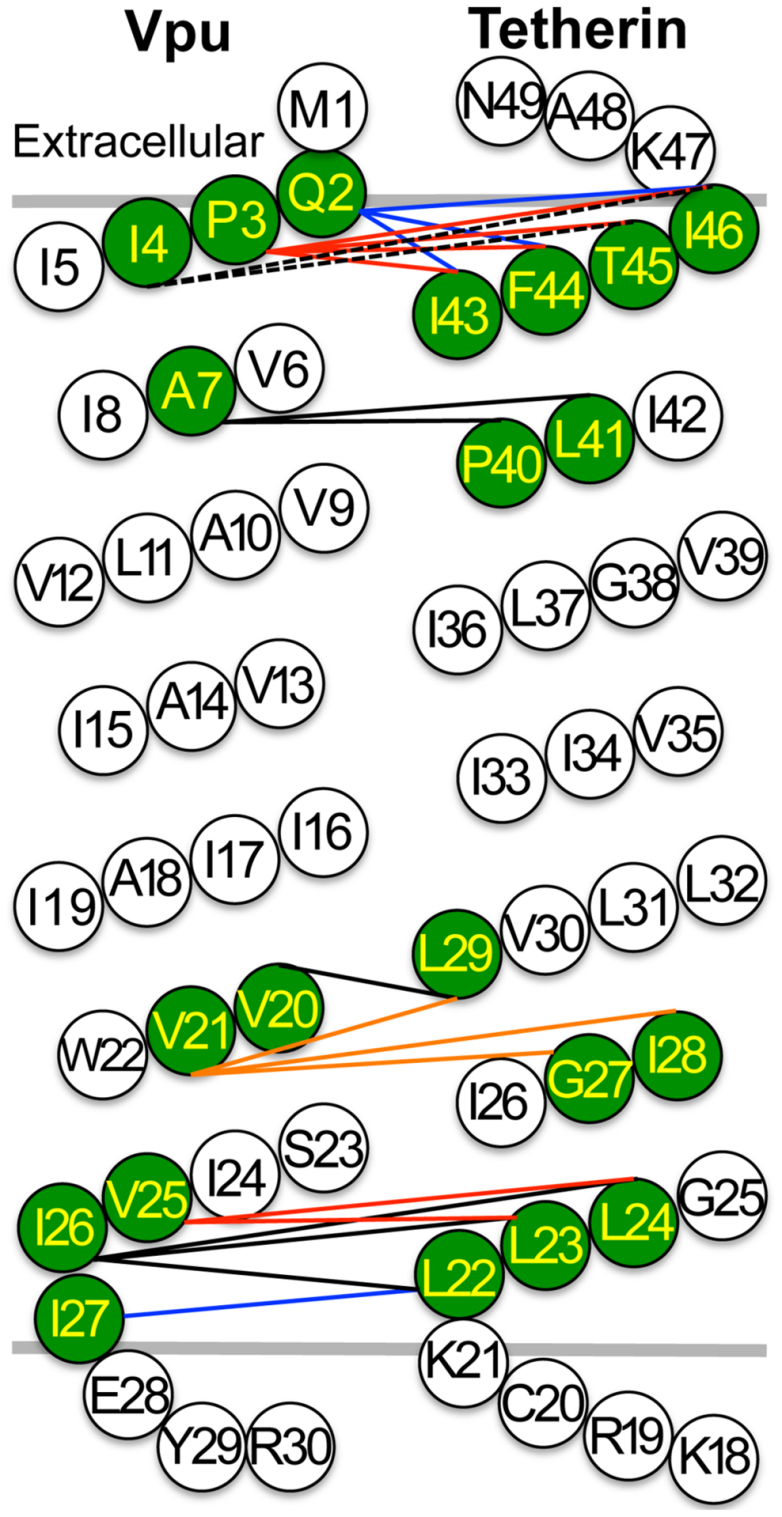
Summary of Vpu and Tetherin TMD residues that formed crosslinks. (**A**) Diagram of Tetherin and Vpu TMDs indicating residues that formed crosslinks, (colored green) and different colored or dotted lines represent the crosslinks between a single Vpu residue and its crosslinked Tetherin residue(s).

**Figure 4:**
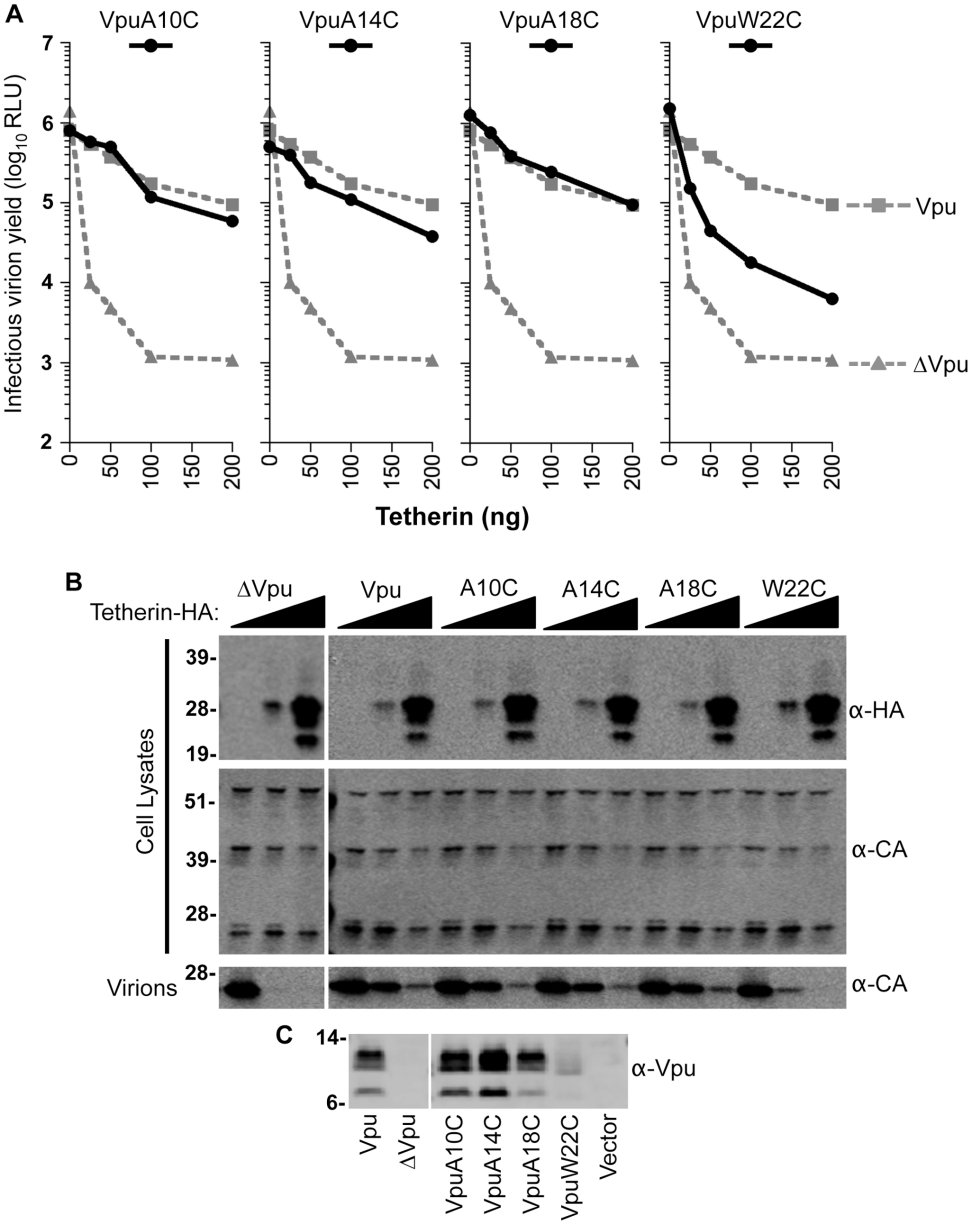
Effect of cysteine substitutions on Vpu activity as determined by virion release assays. (**A**) 293T cells were cotransfected with HIV-1 proviral (pNL4-3) plasmids expressing various mutant Vpu proteins, and with increasing amounts of a plasmid encoding WT Tetherin-HA. Infectious virion yield was measured using TZM indicator cells and is given in relative light units (RLU). (**B**) Western blot analysis of cell lysates and released particles from samples in panel (**A**) that were cotransfected with 0, 25, or 100 ng of Tetherin-HA. Blots of cell lysates were probed with α-HA and capsid α-CA antibodies. Blots of virion lysates were probed with α-CA antibodies. (**C**) Western blot analysis of Vpu expression from samples in panel (**A**) that were transfected with 0 ng of Tetherin. Blots were probed with an α-Vpu antibody.

**Figure 5:**
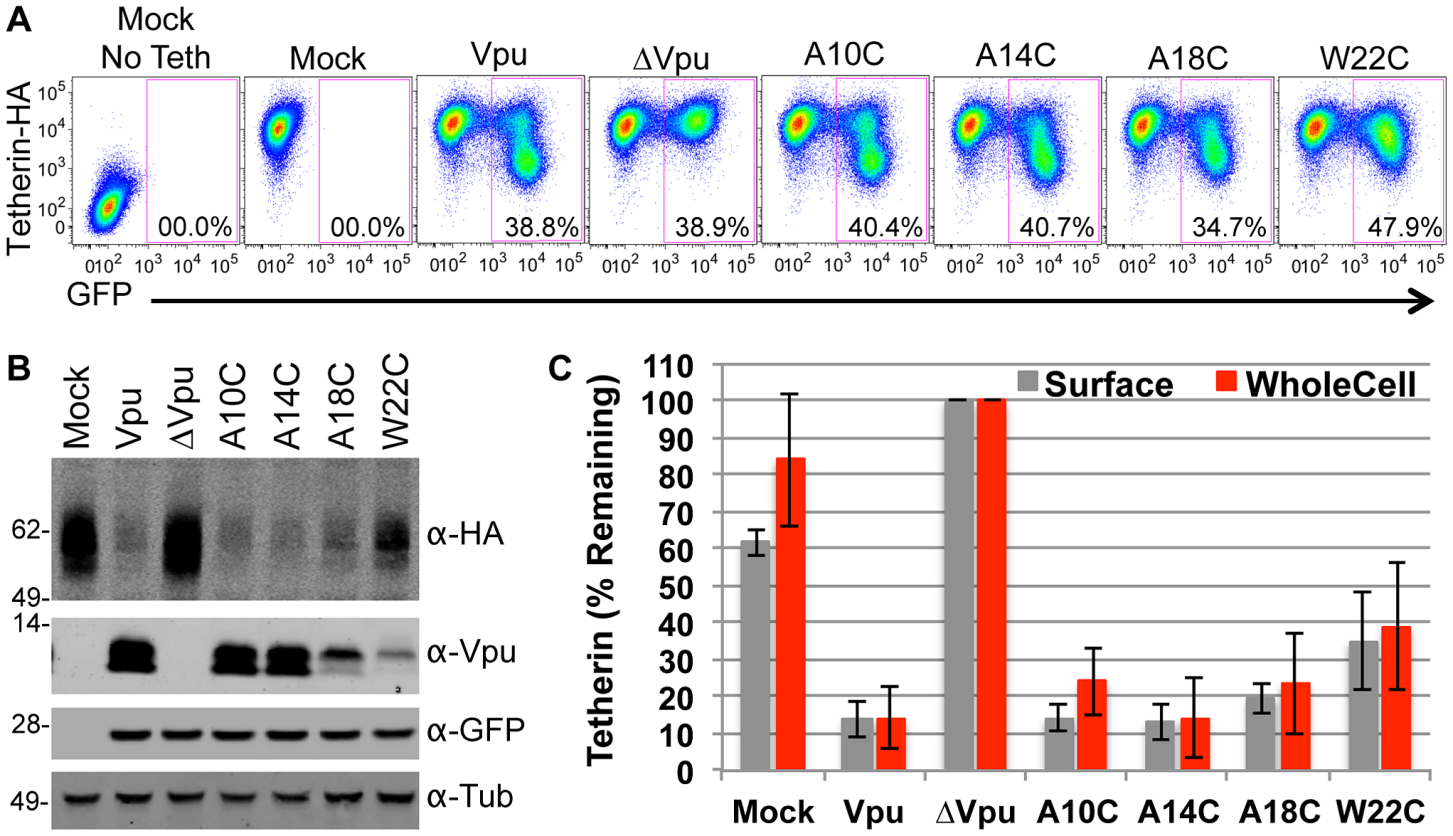
Effect of cysteine substitutions on Vpu activity as determined by Tetherin downregulation and degradation assays. (**A**) 293T cells stably expressing Tetherin-HA were transduced with VSV-G pseudotyped HIV-1 based vectors encoding GFP and various mutant Vpu proteins. Cells transduced at an MOI of 0.5 were surface-stained using an α-Tetherin antibody and analyzed by FACS to determine the relative level of Tetherin-HA expression. (**B**) Cells transduced with the same vectors used in (**A**), but at an MOI of 3, were lysed and analyzed using quantitative fluorescence-based Western blotting. Blots were probed with antibodies specific for Vpu, GFP, tubulin, and HA (Tetherin), as indicated. (**C**) Chart summarizing data from assays carried out as described in (**A**) and (**B**). For FACS analysis, the geometric mean fluorescence after gating on GFP-positive cells was quantified (gray bars). For Western blot analysis band intensities were quantified using a Li-COR scanner (red bars). The mean and standard deviation of the relative amounts of Vpu expression (from four independent experiments) is plotted, with the amount of Tetherin expression observed following transduction with the ΔVpu vector set to 100%.

These results are summarized in Table 1, as a cartoon diagram (Figure 3) and a 3D stereoscopic model (Figure S1) based on NMR-based structural analysis of the Tetherin TMD [44]. Notably, sites of interaction between Vpu and Tetherin, determined by cross-linking, were proximal to some sites that have been reported to be important for the ability of Vpu to antagonize Tetherin and for coimmunoprecipitation [40], [42]–[44], (Table 1). However, cross-linking was not observed at amino acid residues that are predicted to be buried deep within the membrane, including at some sites that have been reported to be important for Tetherin antagonism by Vpu, or for coimmunoprecipitation. For example, Vpu residues A10, A14, A18 and W22 have been reported to be important for Vpu function [43], [44]. However, it was possible that mutating these residues to cysteine for the purposes of our crosslinking experiment disrupted the Vpu-Tetherin interaction and therefore inhibited cross-link formation. To test this hypothesis, we introduced cysteine residues at each of these positions in the context of the HIV-1 proviral plasmid and measured virion release in the presence of Tetherin. Notably, proviral plasmids encoding the Vpu mutants A10C, A14C, and A18C were each capable of generating virions as efficiently as a proviral plasmid encoding WT Vpu (Figure 4A, B). Proviral plasmids encoding the Vpu W22C mutant yielded ∼10-fold fewer virions, however, expression of the VpuW22C mutant was greatly diminished as compared to WT Vpu, which could be responsible for its apparently reduced activity against Tetherin (Figure 4A, B, C). We also tested the ability of this panel of Vpu mutants to downregulate and degrade Tetherin. Again, Vpu mutants A10C, A14C and A18C were indistinguishable from WT Vpu, in these assays, while the poorly expressed VpuW22C mutant was partially able to downregulate and degrade Tetherin (Figure 5A, B, C). We conclude that cysteine mutagenesis of the residues that were previously reported to be important for Vpu function did not, in fact, disrupt Vpu function, except in the case of W22 where expression levels were impaired. We also note that studies in which these residues were identified as important employed an approach in which amino acids with bulky side chains were used to replace alanine. Our cysteine based mutagenesis clearly did not abolish Vpu function, and presumably therefore the ability of Vpu to interact with Tetherin. Therefore, we concluded that these residues either do not contact Tetherin directly, or that the oxidation reagent did not completely penetrate the cell membrane, as has previously been surmised [51].

**Table 1:** Summary of crosslinking and other evidence for Vpu and Tetherin TMD interactions.

VpuTMD				Tetherin TMD				
Amino Acid	Cross-linking	Effects of mutation on Tetherin antagonist function	Other Evidence for interaction with Tetherin	Amino Acid	Cross-linking	Effects of mutation on sensitivity to Vpu	Positive Selection	Other Evidence for interaction with Vpu
Q2	++			I46	++++			
P3	++++			T45	++++	+^b4, c6, d9, e^	+^d, e^	
I4	++++		NMR^c^	F44	+++			
I5	−			I43	+++		+^d^	
V6	−		NMR^c^	I42	−			
A7	+			L41	++	+^b, c^		NMR^c^, IP^c^, BiFC^b^
I8	−			P40	+	+^b(P40A)^	+^d^	
V9	−			V39	−			
A10	−	+^c1,2^	NMR^c^, IP^c^	G38	−			
L11	−			L37	−	+^b, c^		NMR^c^, IP^c^, BiFC^b^
V12	−			I36	−	+^d10,11,12^	+^d, e^	
V13	−			V35	−			
A14	−	+^a, c1,3^	IP^a,c^	I34	−	+^b, c^		NMR^c^, IP^c^, BiFC^b^
I15	−	+^a, c1^	NMR^c^	I33	−	+^d10,11,12^	+^d^	
I16	−			L32	−			
I17	−			L31	−			
A18	−	+^a, c^	NMR^c^, IP^a, c^	V30	−	+^d12, e^	+^d, e^	NMR^c^
I19	−			L29	+++			
V20	++			I28	+	+^d(ΔGI)9,10,13, b(ΔGI)4,5^	+^d^	BiFC^b(ΔGI)^
V21	+			G27	+	+^d(ΔGI), b(ΔGI)^	+^d^	BiFC^b(ΔGI)^
W22	−	+^a, c2,3^	NMR^c^, IP^a, c^	I26	−		+^e^	IP^c^
S23	−		NMR^c^	G25	−	+^c(ΔLG)6,7^		
I24	−			L24	++	+^c(ΔLG),6,7, d13, f(ΔLL)8^	+^d, e^	IP^f^
V25	++++			L23	+++	+^d, f(ΔLL)8^	+^d^	IP^f^
I26	+++		NMR^c^	L22	++			
I27	++		NMR^c^					

References: a [43], b [42], c [44], d [38], e [37], f [40].

Superscript numbers indicate combinations of mutations that were necessary to affect Vpu's Tetherin antagonist function or Tetherin's sensitivity to Vpu.

NMR = Nuclear Magnetic Resonance, BiFC = Bimolecular Fluorescence complementation, IP = Immunoprecipitation.

**Figure 6:**
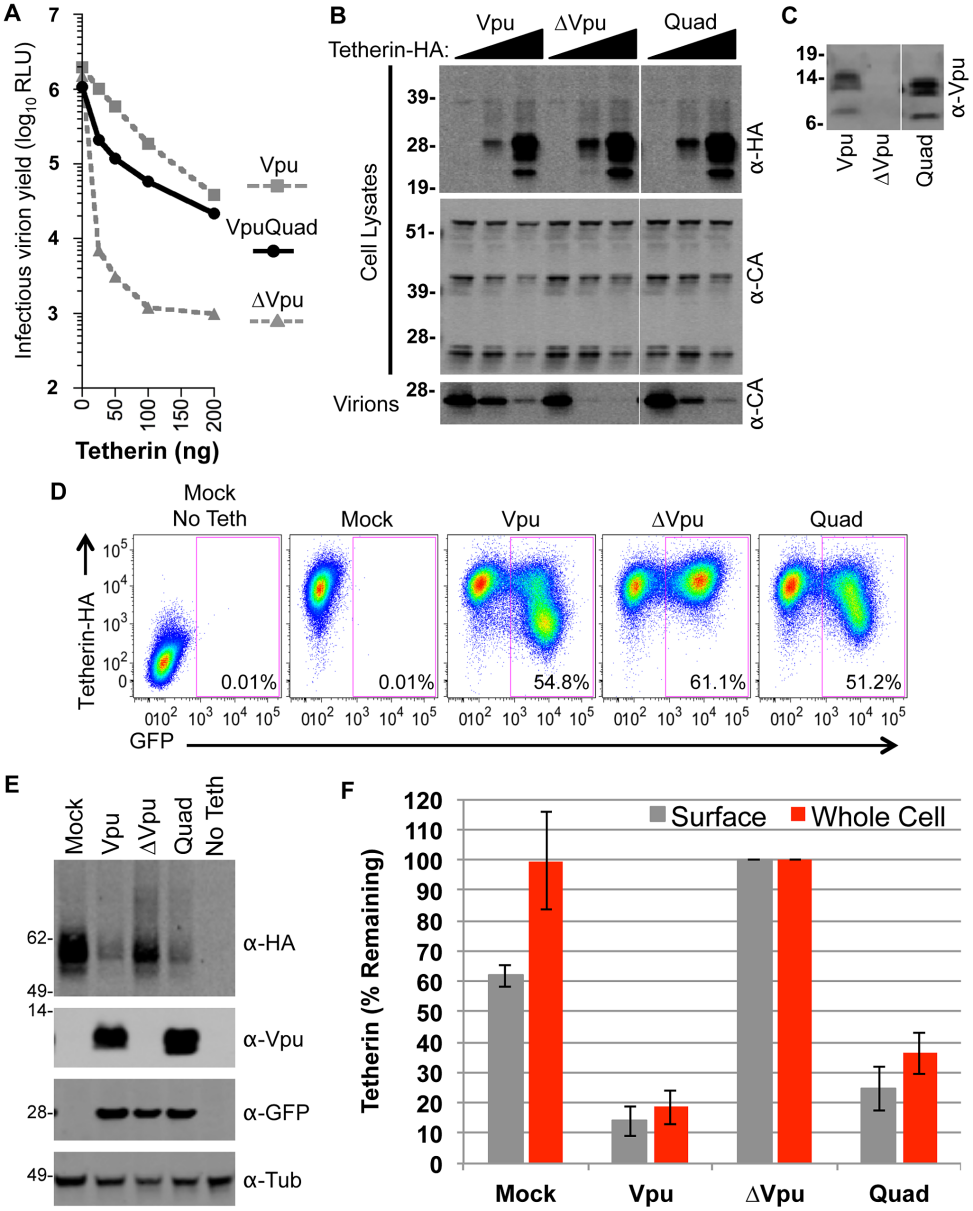
Functional analysis of Vpu mutated at positions that formed efficient crosslinks with Tetherin. (**A**) 293T cells were cotransfected with HIV-1 proviral (pNL4-3) plasmids expressing various mutant Vpu proteins, and with increasing amounts of a plasmid encoding WT Tetherin-HA. Infectious virion yield was measured using TZM indicator cells and is given in relative light units (RLU). (**B**) Western blot analysis of cell lysates and released particles from samples in panel (**A**) that were cotransfected with 0, 25, or 100 ng of Tetherin-HA. Blots of cell lysates were probed with α-HA and capsid α-CA antibodies. Blots of virion lysates were probed with α-CA antibodies. (**C**) Western blot analysis of Vpu expression from samples in panel (**A**) that were transfected with 0 ng of Tetherin. Blots were probed with an α-Vpu antibody. (**D**) 293T cells stably expressing Tetherin-HA were transduced with VSV-G pseudotyped HIV-1 based vector encoding GFP and various mutant Vpu proteins. Cells transduced at an MOI of 0.5 were surface-stained using an α-Tetherin antibody and analyzed by FACS to determine the relative level of Tetherin-HA expression. The FACS plots show examples of this assay. (**E**) Cells transduced with the same vectors used in (**D**), but at an MOI of 3, were lysed and analyzed using quantitative fluorescence-based Western blotting. Blots were probed with antibodies specific for Vpu, GFP, tubulin, and HA, as indicated. (**F**) Chart summarizing data from assays carried out as described in (**D**) and (**E**). For FACS analysis, the geometric mean fluorescence after gating on GFP-positive cells was quantified (gray bars). For Western blot analysis band intensities were quantified using a LI-COR scanner (red bars). The mean and standard deviation of the relative amounts of Vpu expression (from four independent experiments) is plotted, with the amount of Tetherin expression observed following transduction with the ΔVpu vector set to 100%.

While residues in the center of the membrane embedded helices may have been inaccessible to the crosslinking reagent, these experiments nevertheless revealed four major points of contact between Vpu and Tetherin, centered on Vpu residues I4, A7, V20, and I26 (Figures 2 and 3). To determine whether these contact points were required for Vpu function, we mutated four residues in Vpu (I4A, A7L, V20A, and I26A) generating a mutant termed VpuQuad. This approach was taken because it has often been found that more than one Vpu mutation is necessary to inhibit Tetherin antagonism (Table 1). First, the VpuQuad mutant was expressed in the context of a HIV-1 proviral plasmid, in the presence of WT Tetherin. Surprisingly, Vpu Quad was expressed at higher levels as compared to WT Vpu but was nevertheless partially defective as compared to WT Vpu in virion release assays, and a provirus encoding the VpuQuad mutant yielded approximately 5-fold fewer infectious virions in the presence of Tetherin (Figure 6A, B, C). Concordantly, the VpuQuad mutant displayed a partial defect in its abilities to downregulate and degrade Tetherin (Figure 6D, E, F).

In conclusion, even though this crosslinking approach indicates that there are multiple sites of contact between Vpu and Tetherin TMDs, it likely underestimates the full extent of the interaction, perhaps because of the inability of the oxidizer to penetrate the membrane. Overall it appears that the interaction between Vpu and Tetherin TMDs is extensive. Indeed, even mutation of four amino acids that can be demonstrated to be in close proximity to Tetherin in Vpu-Tetherin complexes resulted in a mutant Vpu (VpuQuad) whose activity was only partly attenuated. Nonetheless, these analyses clearly demonstrate that Vpu and Tetherin TMDs bind directly to each other at several points, and in a physiological context (i.e. a cell membrane).

### Multiple domains in the Vpu cytoplasmic tail contribute to Tetherin antagonism

**Figure 7:**
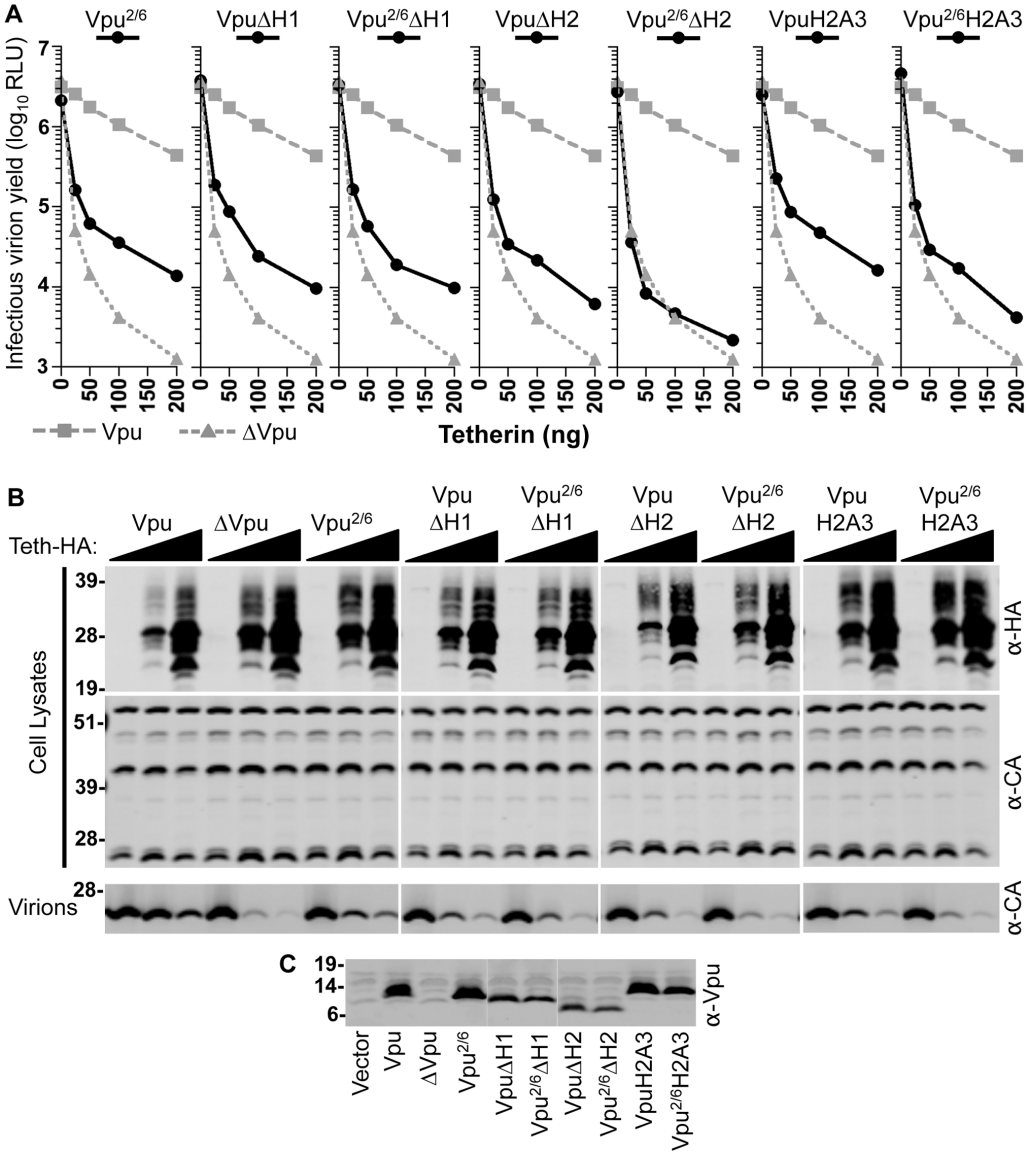
Multiple elements in the Vpu CTD are required to fully antagonize Tetherin. (**A**) 293T cells were cotransfected with HIV-1 proviral (pNL4-3) plasmids expressing various mutant Vpu proteins, and with increasing amounts of a plasmid encoding WT Tetherin-HA. Infectious virion yield was measured using TZM indicator cells and is given in relative light units (RLU). (**B**) Western blot analysis of cell lysates and released particles from samples in panel (**A**) that were cotransfected with 0, 25, or 100 ng of Tetherin-HA. Blots of cell lysates were probed with α-HA and capsid α-CA antibodies. Blots of virion lysates were probed with α-CA antibodies. (**C**) Western blot analysis of Vpu expression from samples in panel (**A**) that were transfected with 100 ng of Tetherin. Blots were probed with an α-Vpu antibody.

The binding of the Vpu TMD to the Tetherin TMD appears necessary for Vpu function, but may serve simply to recruit the cytoplasmic domain of Vpu to the vicinity of Tetherin. Indeed, elements of the Vpu CTD have been shown to be important determinants of Tetherin antagonist activity, including residues between the TMD and H1 [26], H2 [26], an ExxxLV motif in H2 [50], and the two phosphorylatable serines (mutated in Vpu^2/6^) that are known to be required for β-TrCP recruitment [25] and for Tetherin downregulation [2], [28]–[31], [33]–[36] and degradation [28], [31], [33]–[36]. Therefore, we next sought to determine how each of the aforementioned elements of the Vpu CTD contribute Tetherin antagonist function. Consistent with some previous observations [28], [33], [34], [49], we found that the Vpu^2/6^ mutant retained partial Tetherin antagonist activity (Figure 7A, B), and was expressed at similar levels to WT Vpu (Figure 7C). Indeed, assessment of a panel of Vpu cytoplasmic tail mutants suggested that multiple elements in the Vpu CTD contribute to Tetherin antagonist function (Figure 7A, B, C). Specifically, Vpu mutants bearing deletions of H1 (VpuΔH1) or H2 (VpuΔH2), and mutants bearing a mutation of the β-TrCP binding site (Vpu^2/6^) or the H2 ExxxLV motif to AxxxAA (VpuH2A3) all exhibited residual ability to antagonize Tetherin (Figure 7A). Each of these Vpu mutants, expressed in the context of an HIV-1 proviral plasmid, was impaired as compared to WT Vpu, exhibiting about 10% of WT Vpu activity, but each was able to increase the yield of infectious HIV-1 virions by ∼10 fold, as compared to a ΔVpu proviral plasmid (Figure 7A). Some combinations of mutations (e.g. combining the H1 deletion with the β-TrCP binding site mutation (in Vpu^2/6^ΔH1)) resulted in a Vpu protein that retained the same residual activity of each of the individual mutants (Figure 7A). In contrast, combining, for example, the H2 deletion with the β-TrCP binding site mutation (in Vpu^2/6^ΔH2) resulted in a Vpu protein that was completely inactive (Figure 7A), even though, Vpu^2/6^ΔH2 and VpuΔH2 were expressed at the same level (Figure 7C). Combining the H2A3 motif mutation with the Vpu^2/6^ binding mutation (in Vpu^2/6^H2A3) resulted in a Vpu protein with reduced activity as compared to the VpuH2A3 or the Vpu^2/6^ mutants. In general, these data suggest that the Vpu CTD contains multiple elements that contribute to overall Tetherin antagonist function.

**Figure 8:**
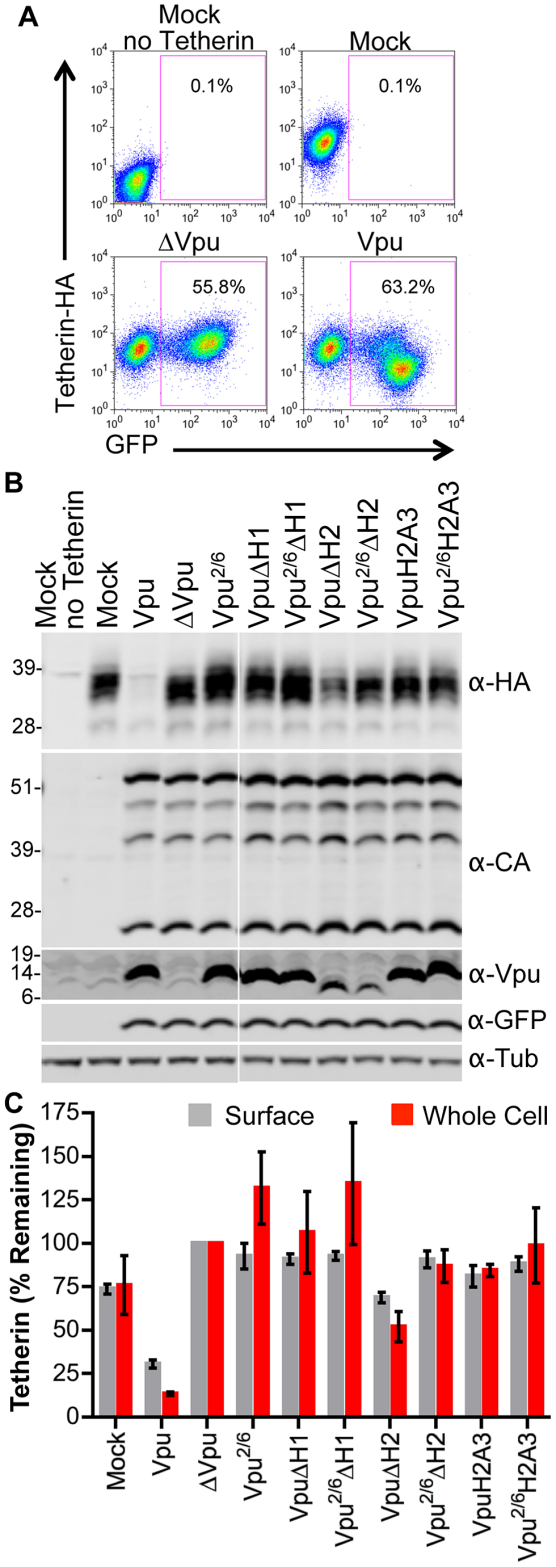
Multiple Vpu CTD elements are required for optimal Tetherin downregulation and degradation. (**A**) 293T cells stably expressing Tetherin-HA were transduced with VSV-G pseudotyped HIV-1 based vector encoding GFP and various mutant Vpu proteins. Cells transduced at an MOI of 0.5 were surface-stained using an α-HA antibody and analyzed by FACS to determine the relative level of Tetherin-HA expression. The FACS plots show examples of this assay. (**B**) Cells transduced with the same vectors used in (**A**), but at an MOI of 3, were lysed and analyzed using quantitative fluorescence-based Western blotting. Blots were probed with antibodies specific for Vpu, GFP, tubulin, capsid and HA, as indicated. (**C**) Chart summarizing data from assays carried out as described in (**A**) and (**B**). For FACS analysis, the geometric mean fluorescence after gating on GFP-positive cells was quantified (gray bars). For Western blot analysis band intensities were quantified using a LI-COR scanner (red bars). The mean and standard deviation of the relative amounts of Vpu expression (from four independent experiments) is plotted, with the amount of Tetherin expression observed following transduction with the ΔVpu vector set to 100%.

To determine the role of Vpu CTD domains in degradation of Tetherin and/or downregulation of Tetherin from the cell surface, viruses expressing a panel of Vpu mutants were assessed for these two functions in infected cells (Figure 8A, B, C). As expected, WT Vpu induced surface downregulation and degradation of Tetherin (Figure 8A, B). In contrast, all of the Vpu CTD mutants were either devoid of Tetherin surface downregulation/degradation activity, or impaired in their ability to mediate these effects (Figure 8B, C). This suggested that multiple elements within the CTD, or perhaps the overall protein structure of the Vpu CTD are important for Tetherin downregulation and degradation. However, the VpuΔH2 mutant retained some ability to induce Tetherin downregulation and degradation suggesting that the H2 domain is not absolutely required for these activities (Figure 8B, C). Nonetheless the H2 domain and the ExxxLV motif within it clearly contributed to Tetherin antagonist activity (Figure 7A).

### Vpu binding displaces Tetherin from sites of viral assembly in the absence of downregulation or degradation

**Figure 9:**
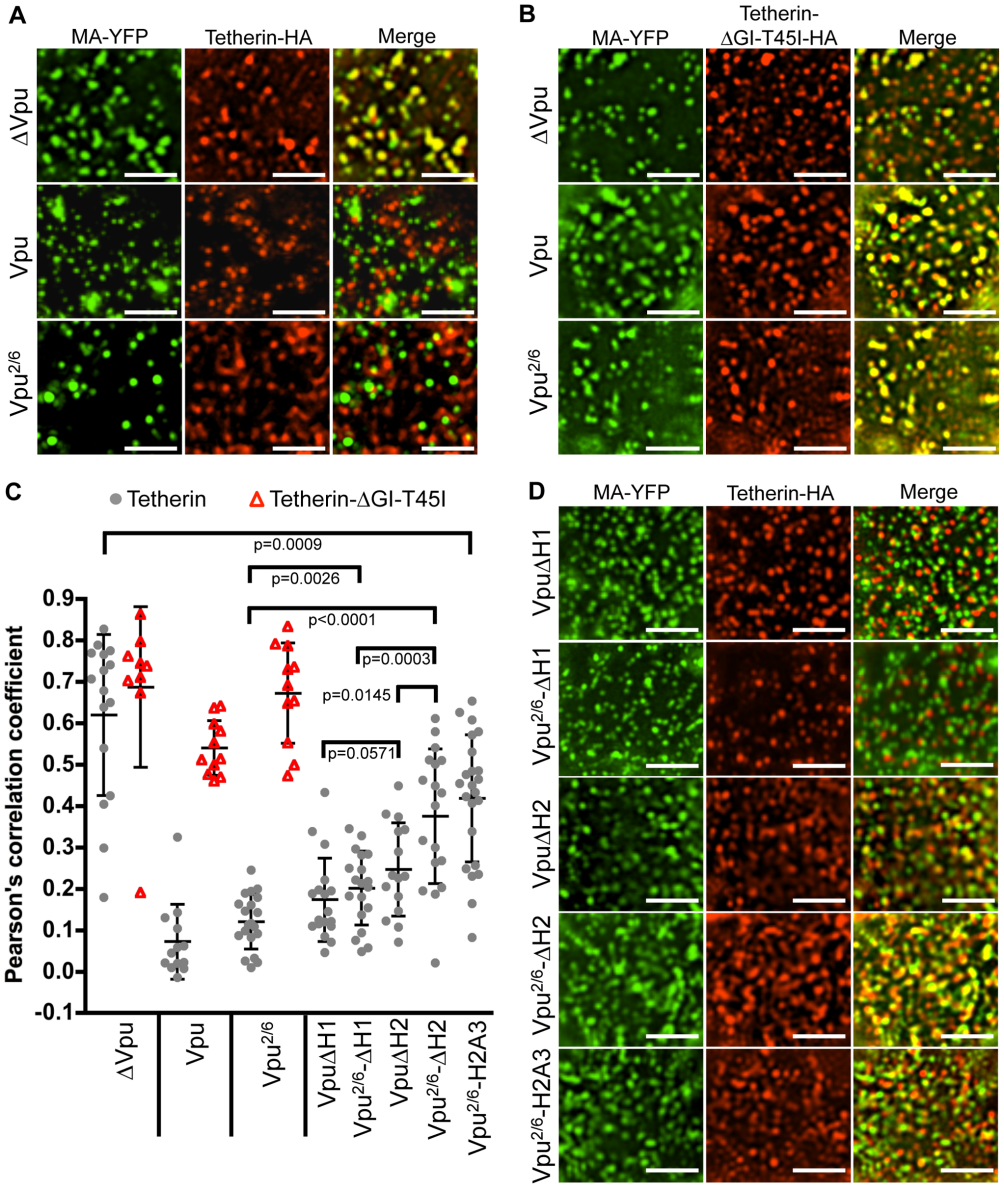
Vpu-induced displacement of Tetherin from sites of viral assembly. (**A, B and D**) 293T cells stably expressing Tetherin-HA were transfected with HIV-1 proviral pNL4-3 plasmids encoding an MA-YFP-fusion protein (green) to label virions. The Vpu protein was either unaltered or mutated as indicated. Fixed, non-permeabilized cells were stained with α-HA to reveal surface Tetherin-HA protein (Alexa-594, red). Scale bars represents 3 µm. (**C**) A graph summarizing the quantification of colocalization at the PM between virions and WT Tetherin-HA or TetherinΔGI-T45I-HA. Each symbol represents the Pearson's correlation coefficient for an individual cell, and horizontal lines and error bars represent means and standard deviations for populations of cells. Colocalization was quantified in multiple regions of interest on the dorsal surface of each cell. The indicated p values were calculated using Student's two-tailed unpaired t test.

Notably, some of the Vpu mutants (for example Vpu^2/6^, VpuΔH1, Vpu^2/6^ΔH1) appeared to completely lack the ability to induce Tetherin surface downregulation and degradation (Figure 8), yet retained some ability to antagonize Tetherin activity (Figure 7). To determine how Vpu impairs Tetherin activity in the absence of downregulation and degradation, we examined the distribution of cell surface Tetherin in cells in which fluorescently labeled HIV-1 particles were assembling. As we and others have previously shown [4], [19], [52], Tetherin colocalized with virions at the plasma membrane (PM) in the absence of Vpu (Figure 9A). Conversely, Tetherin-virion colocalization was inhibited in the presence of WT Vpu (Figure 9A). The TetherinΔGI-T45I mutant, which exhibits reduced ability to bind Vpu (Figure 1A and 1D), colocalized with virions even in the presence of Vpu, consistent with the notion that displacement of Tetherin from nascent virions at the cell surface is dependent on binding between the Vpu and Tetherin TMDs (Figure 9B). These data are quantified and are summarized in Figure 9C.

Notably, the Vpu^2/6^ mutant, which was devoid of Tetherin downregulation and degradation activity, also specifically inhibited WT Tetherin but not TetherinΔGI-T45I colocalization with virions (Figure 9A, B, lower row of panels). Analysis of a wider panel of Vpu CTD mutants revealed that VpuΔH1, and Vpu^2/6^ΔH1 mutants retained full ability to inhibit colocalization of Tetherin with nascent virions. These Tetherin mutants each lacked downregulation and degradation activity, but notably, encoded an intact H2 domain (Figure 9C, D). The ability of Vpu to inhibit colocalization of Tetherin with virions was reduced slightly by the VpuΔH2 mutation (Figure 9C, D), but it is important to note that this mutant is also capable of promoting Tetherin downregulation and degradation (Figure 8B, C). However, when Tetherin downregulation and degradation was also abrogated by inclusion of the Vpu^2/6^ mutation (in Vpu^2/6^ΔH2), this protein was only poorly able to displace Tetherin from sites of viral assembly (Figure 9C, D). The Vpu^2/6^H2A3 mutant was even less able to displace Tetherin from sites of viral assembly compared to Vpu^2/6^ΔH2 (Figure 9D). Thus, these data suggest that the Vpu H2 domain, and the ExxxLV motif within it, was important for the displacement of Tetherin from sites of viral assembly. Moreover, the ability of Vpu mutants to displace Tetherin from sites of virion assembly may explain how they are able to partially antagonize Tetherin in the absence of Tetherin downregulation or degradation.

### Artificial recruitment Vpu CTD elements to Tetherin can recapitulate Tetherin antagonism in the absence of TMD interactions

**Figure 10:**
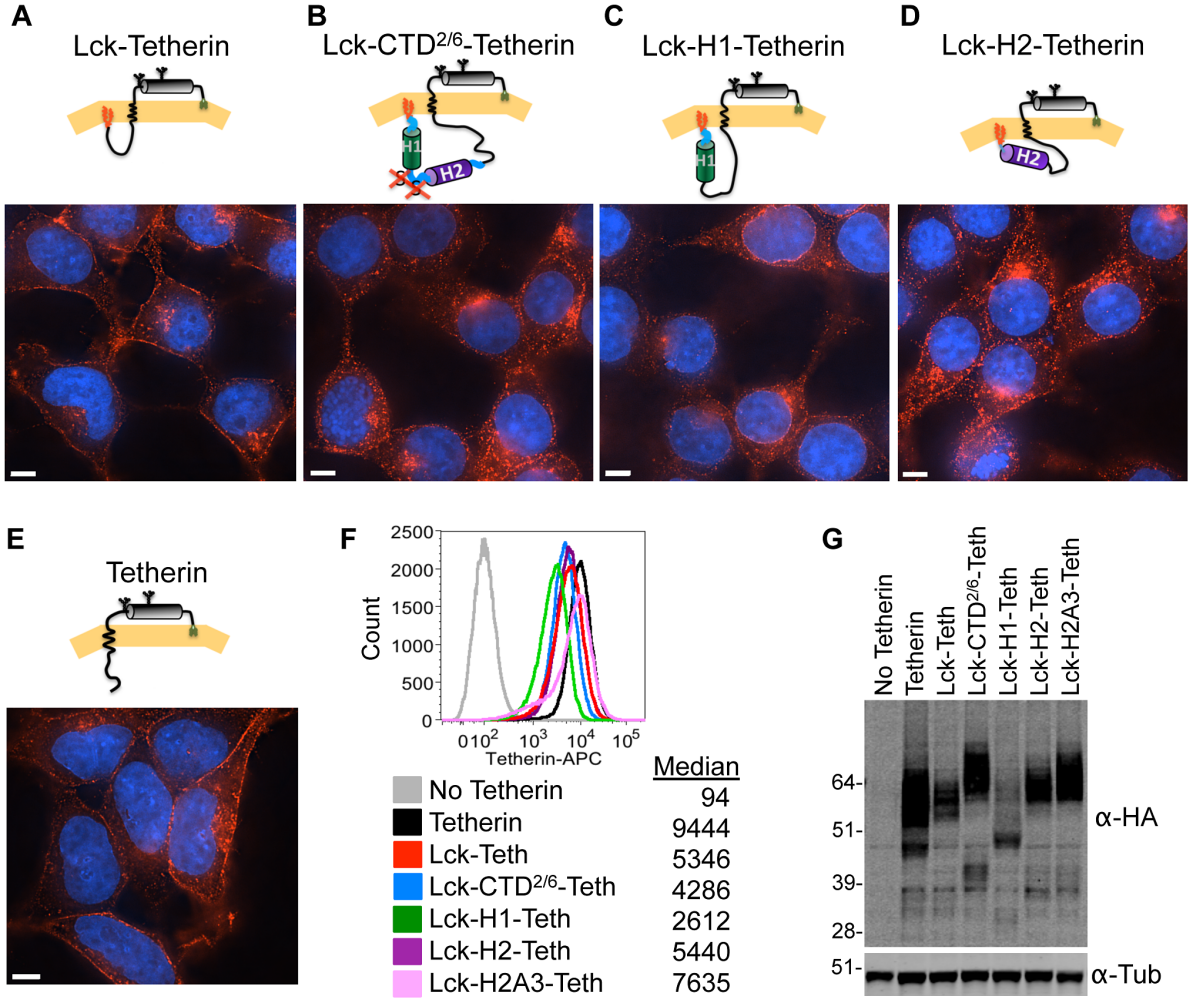
Localization of Lck-Vpu-Tetherin-HA fusion proteins. (**A**–**E**) Single cell clones of 293T cells stably expressing the indicated proteins namely: Lck-Tetherin (**A**), Lck-CTD^2/6^-Tetherin (**B**), Lck-H1-Tetherin (**C**), Lck-H2-Tetherin (**D**) or WT Tetherin (**E**) were fixed, permeabilized and stained using α-HA antibodies (Alexa-594, red) and with DAPI to reveal nuclei (blue). Scale bar represents 5 µm. (**F**) Single cell clones stably expressing proteins from (**A–E**) were cell surface stained using an antibody against Tetherin directly conjugated to the flourochrome APC. Living and singlet cells were then gated and the Tetherin signal was reported as the median fluorescent intensity. (**G**) Western blot analysis of cell lysates from single cell clones expressing various forms of Tetherin as described in (**A–E**).

While the Vpu TMD is necessary for Tetherin binding, the above data indicate that various additional Vpu CTD sequences are also required for Tetherin antagonism, and the displacement of Tetherin from virions. To determine whether Vpu TMD–Tetherin TMD interactions are necessary for the ability of Vpu to displace Tetherin from nascent virions, or simply provide a means by which Vpu is recruited to Tetherin, we adopted an experimental strategy in which the Vpu CTD was artificially recruited to Tetherin in the absence of the TMD. Specifically, we engineered proteins in which either the entire Vpu CTD, or individual elements thereof, were fused to the N-terminus of Tetherin (Figure 10A–E). An additional advantage of this approach is that it ensures that every Tetherin molecule is associated with a Vpu CTD or CTD fragment at a stoichiometric ratio of 1∶1. To retain the topological relationship of the Vpu CTD with the cell membrane, we appended its N-terminus with a sequence of amino acids (MGCGCSSHPEGGG) from the N-terminus of Lck, generating a palmitoylated and myristoylated membrane anchor (Figure 10A–D) [53]–[55]. Transient expression of the modified Tetherin proteins revealed that each was expressed at the cell surface (Figure S2A). Indeed, transiently expressed WT Tetherin, Lck-Tetherin and Lck-H2-Tetherin, were present on at the cell surface at similar levels (Figure S2A). However, both Lck-CTD^2/6^-Tetherin and especially Lck-H1-Tetherin were expressed at the cell surface at ∼3-fold and ∼5-fold lower levels respectively (Figure S2A).

To facilitate microscopic analysis of the localization of the Tetherin proteins we generated clonal cell lines that expressed each of the modified Tetherin proteins at similar levels (insofar as was possible) at the cell surface (Figure 10F). In this panel of cell lines, the levels of the Lck-Tetherin proteins were more modest than in transiently transfected cells, and quite well matched, with the exception of the Lck-H1-Tetherin, which only yielded clones expressing lower levels of Tetherin at the cell surface. Microscopic examination of these cell lines revealed that when the Tetherin N-terminus was appended with the Lck N-terminus alone, (in Lck-Tetherin, Figure 10A) The modified Lck-Tetherin protein exhibited a subcellular localization that was indistinguishable from the unmodified Tetherin protein (Figure 10E).

The Lck-Tetherin protein that was appended with the H2 domain of Vpu CTD (Lck-H2-Tetherin, Figure 10D) exhibited a subcellular localization that was similar to WT Tetherin or Lck Tetherin, with prominent cell surface staining. In contrast, the Tetherins appended with the entire Vpu CTD^2/6^ (Lck-CTD^2/6^-Tetherin, Figure 10B), or particularly the H1 domain alone (Lck-H1-Tetherin, Figure 10C) were less prominently localized to the cell surface. Indeed, the Lck-H1-Tetherin fusion protein appeared to localize in part in a perinuclear ring (Figure 10C) and colocalized with a transiently expressed a DsRED-ER marker (Pearson's coefficient = 0.6) whereas, the Lck-Tetherin protein exhibited only minimal colocalization with the ER marker (Pearson's coefficient = 0.02) (Figure S2B). Consistent with the notion that H1 induces ER retention, western blot analysis of the clonal stable cell lines expressing the Lck-Tetherin proteins revealed that Lck-H1-Tetherin dimers were of lower molecular weight than that the other Tetherin proteins, presumably because they failed to acquire the high molecular weight complex carbohydrates that are characteristic of Tetherin proteins that have proceeded through the secretory pathway [14]. These data are also consistent with previous observations demonstrating that a Vpu protein harboring a C-terminal truncation can modify the subcellular localization of Tetherin [26]. Overall these findings suggest the presence of an ER membrane retention activity in H1. However, this property was less evident in the Lck-CTD^2/6^-Tetherin (Figure 10B), suggesting the possibility that H2 suppresses the putative ER retention signal in H1.

**Figure 11:**
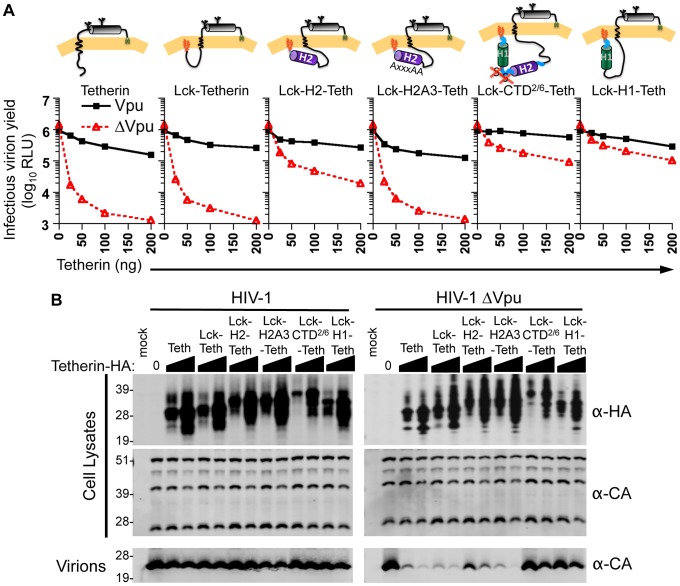
Fusion of Vpu CTD fragments to Tetherin can recapitulate Vpu function. (**A**) 293T cells were cotransfected with HIV-1 WT Vpu or HIV-1 ΔVpu proviral (pNL4-3) plasmids and with increasing amounts of the indicated Lck-Vpu-Tetherin-HA expression plasmids as depicted above each graph. Infectious virion yield from transfected cells was measured using HeLa-TZM indicator cells and is given in relative light units (RLU). (**B**) Western blot analysis of cell lysates and released particles from cells transfected with 0, 50 and 200 ng of the Tetherin fusion proteins from panel (**A**). Cell lysates and virions were probed with α-HA and/or α-CA antibodies.

Not only did the modified Lck-Tetherin protein (Figure 10A) exhibit a subcellular localization that was indistinguishable from the unmodified Tetherin protein (Figure 10E), but it also restricted virion release, and retained Vpu sensitivity (Figure 11A, B). Thus, the Lck modification was, by itself, apparently inert. Analyses of virion release in the presence of the various Lck-Vpu-Tetherin fusion proteins revealed that the entire CTD^2/6^, as well as the individual H1 or H2 domains, were able to antagonize the antiviral activity of Tetherin when fused to its N-terminus (Figure 11A, B). The inability of Lck-H1-Tetherin to inhibit HIV-1 particle release might be explained by the redistribution of the modified Tetherin protein away from the PM (Figures 10, Figure S2). However, Lck-H2-Tetherin was abundantly expressed at the PM (Figure 10, Figure S2), suggesting that Vpu H2 harbors an activity that antagonizes Tetherin, without removing it from the PM (Figure 11A, B). The apparent ability of H2 to antagonize Tetherin in the context of the Lck-Tetherin fusion protein was abolished when ExxxLV motif in H2 was mutated to AxxxAA in Lck-H2A3-Tetherin, (Figures 11A, 11B, and S2). Thus, these data demonstrate that the Vpu H2 domain and ExxxLV motif within it contribute to Vpu function, in the absence of Tetherin downregulation and degradation.

### Vpu H2 is sufficient to displace Tetherin from nascent virions

**Figure 12:**
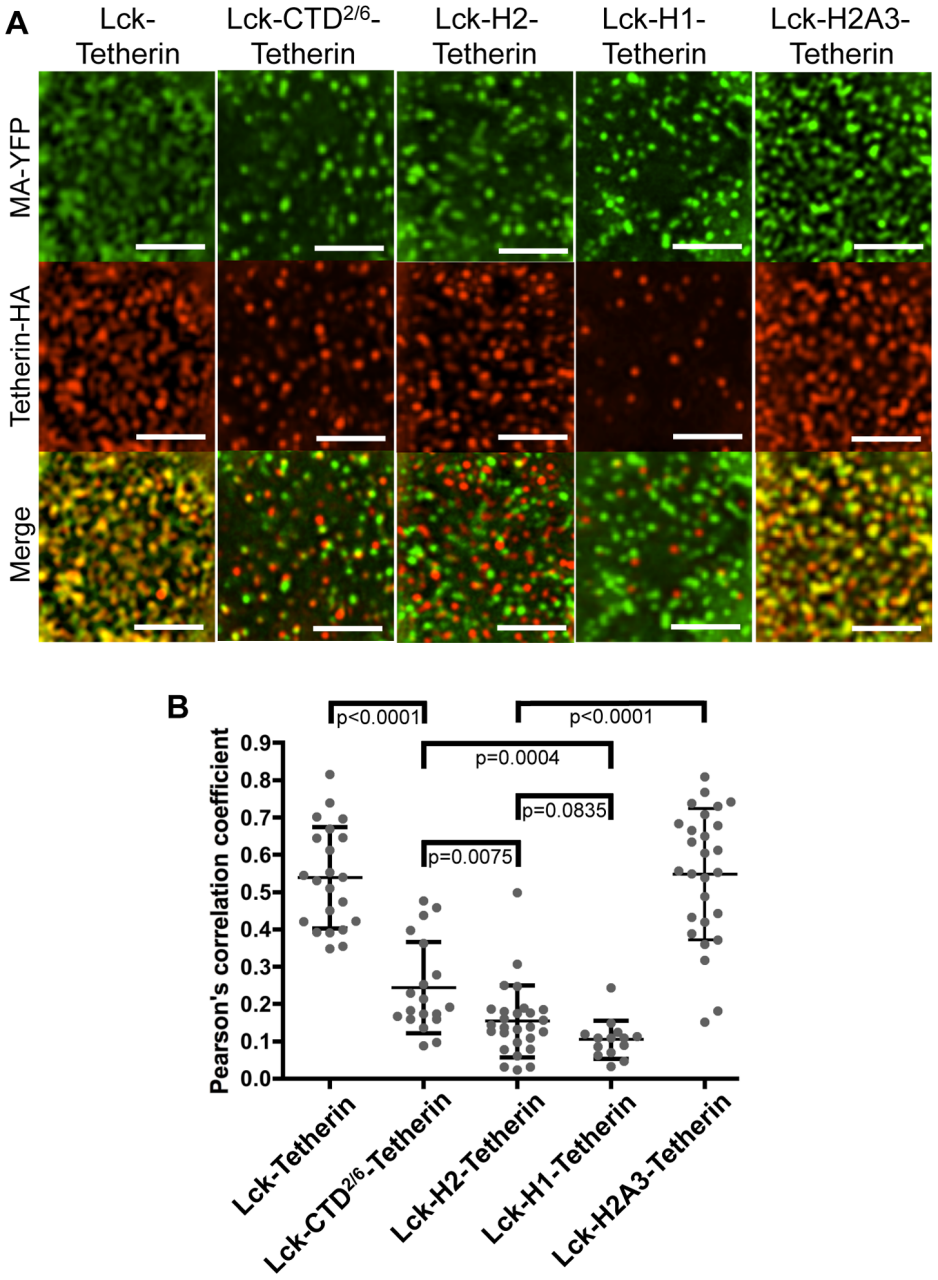
Fusion of Vpu CTD fragments to Tetherin can displace the protein from virions. (**A**) 293T cell lines stably expressing Lck-Vpu-Tetherin-HA fusion proteins (as in Figure 10) were transfected with HIV-1 ΔVpu proviral plasmids encoding an MA-YFP-fusion protein to label virions (green). Fixed, non-permeabilized cells were stained with α-HA to reveal surface Tetherin-HA protein (Alexa-594, red) as in Figure 9. Scale bar represents 3 µm and merged images are shown in the bottom row. (**B**) The graph summarizes the quantification of colocalization between virions and Lck-Tetherin-HA fusion proteins. Each symbol represents the Pearson's correlation coefficient for an individual cell, and horizontal lines and error bars represent means and standard deviations of populations of cells. The indicated p values were calculated using Student's two-tailed unpaired t test.

To determine whether the isolated Vpu CTD and fragments thereof could modulate the colocalization of Tetherin with nascent virions, we used fluorescence microscopy to quantify the colocalization between the Lck-Vpu-Tetherin fusion proteins and fluorescently labeled HIV-1 particles. As compared to Lck-Tetherin, the Lck-CTD^2/6^-Tetherin fusion protein was impaired in its ability to colocalize with virions, (Figure 12A, B). Moreover, Lck-Tetherin constructs encoding either H1 or H2 fragments alone (Lck-H1-Tetherin and Lck-H2-Tetherin) appeared to be nearly completely excluded from virions (Figure 12A,B). Note again, the Lck-H1-Tetherin protein was primarily localized to internal compartments, and comparatively poorly expressed on the cell surface, which may be partly responsible for the exclusion of Lck-H1-Tetherin from nascent virions. Conversely, Lck-H2-Tetherin is abundant on the cell surface, but did not colocalize with nascent virions (Figure 10, Figure S2, Figure 12A, B). Notably, mutating the ExxxLV motif within H2 abolished the ability of H2 to cause exclusion of Lck-Tetherin from nascent virions (Figure 12A, B). Thus, these experiments demonstrate that the isolated Vpu H2 domain is sufficient to displace Tetherin from sites of viral assembly, in the context of Lck-H2-Tetherin, even when the fusion protein is abundantly expressed at the cell surface. Interestingly, the isolated H1 and H2 domains were slightly more potent than the complete CTD^2/6^ in inhibiting Lck-Tetherin colocalization with virions, but both were clearly and independently able to inhibit Tetherin colocalization with virions and inhibit antiviral activity (Figures 11 and 12).

**Figure 13:**
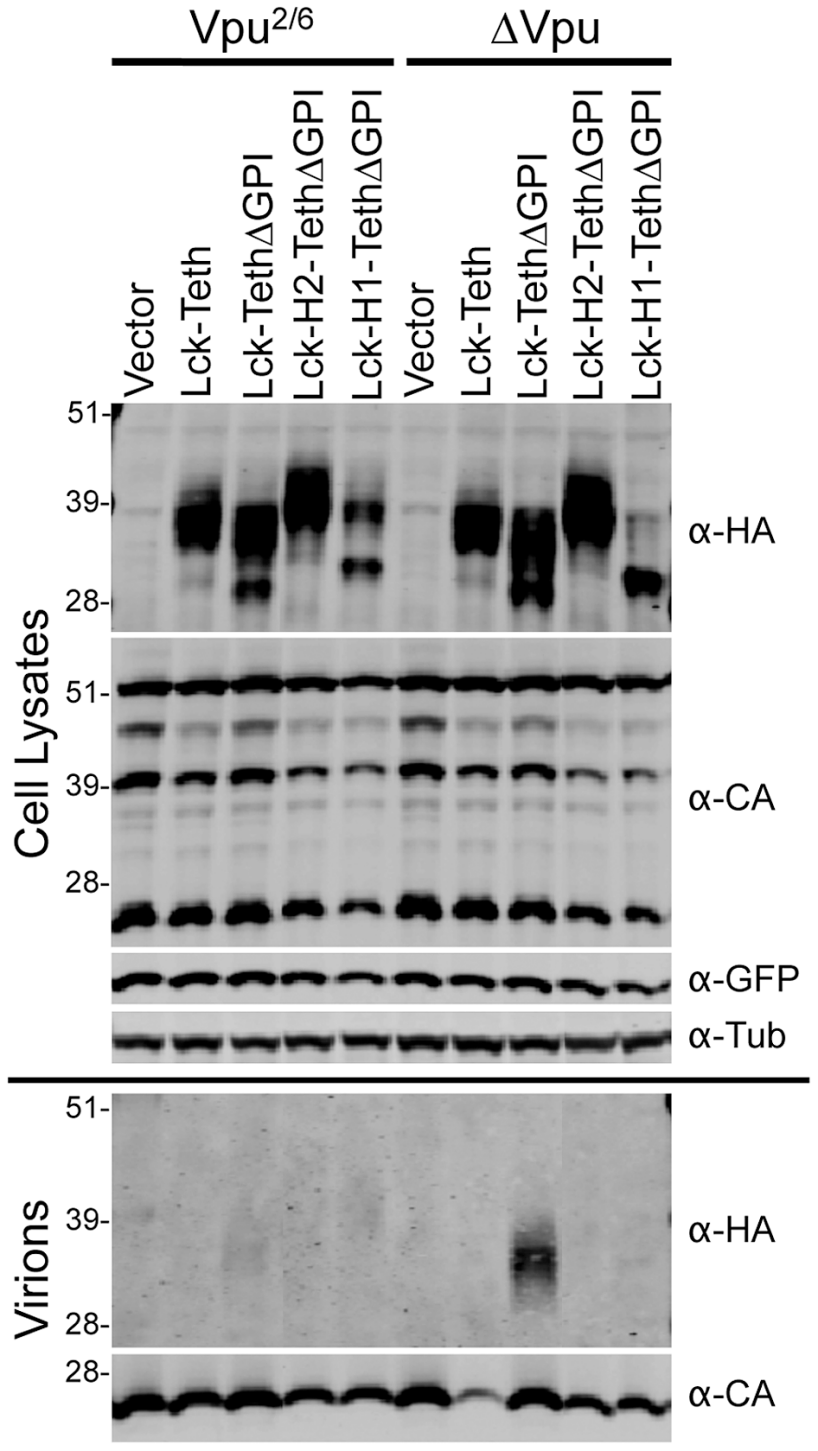
Fusion of VpuH2 to Lck-TetherinΔGPI blocks its incorporation into virions. 293T cells stably expressing HA-tagged Lck-Tetherin, Lck-TetherinΔGPI, Lck-H2-TetherinΔGPI, or Lck-VpuH1-TetherinΔGPI fusion proteins were infected with VSV-G pseudotyped HIV-1ΔNef-GFP virus encoding either no Vpu (ΔVpu) or the Vpu^2/6^ protein. Thereafter, cell lysates and isolated progeny virions were subjected to Western blot analyses with α-CA, α-HA, α-GFP, and α-tubulin antibodies, as indicated. The migration of markers of the indicated molecular weights (kDa) is indicated left of the blots.

To further investigate this apparent displacement activity, we assessed the ability of the Vpu CTD fragments to block incorporation of Tetherin into released virion particles. This strategy was based on our previous finding that removal of the GPI anchor from Tetherin (TetherinΔGPI) causes a loss of antiviral activity and efficient incorporation of the TetherinΔGPI protein into released virions [14]. In this experiment, we stably expressed Lck-Tetherin, Lck-TetherinΔGPI, Lck-H2-TetherinΔGPI, or Lck-H1-TetherinΔGPI and infected cells with viruses that encoded Vpu^2/6^ or lacked Vpu (ΔVpu). All cells that expressed Vpu^2/6^ efficiently released virions and did not efficiently incorporate Lck-TetherinΔGPI protein into virions (Figure 13). As before, Lck-Tetherin inhibited virus release in the absence of Vpu (Figure 11A, B, Figure 13). Conversely, cells expressing Lck-TetherinΔGPI generated similar amounts of released virions as did unmodified cells. Importantly, Lck-TetherinΔGPI was efficiently incorporated into virions, specifically in the absence of Vpu (Figure 13). Further, both the Vpu H1 and H2 fragments directly appended to Tetherin (in the context of a Lck-TetherinΔGPI protein) could inhibit Lck-TetherinΔGPI incorporation into released virions (Figure 13). As before, Tetherin localization to internal compartments was induced by H1, likely contributed to the lack of incorporation of Lck-H1-TetherinΔGPI into virions, while Lck-H2-TetherinΔGPI was well expressed at the cell surface (Figure S3). Therefore, H2, when directly appended to TetherinΔGPI, inhibited the incorporation of Tetherin into virions without affecting levels of cell surface expression.

## Discussion

**Figure 14:**
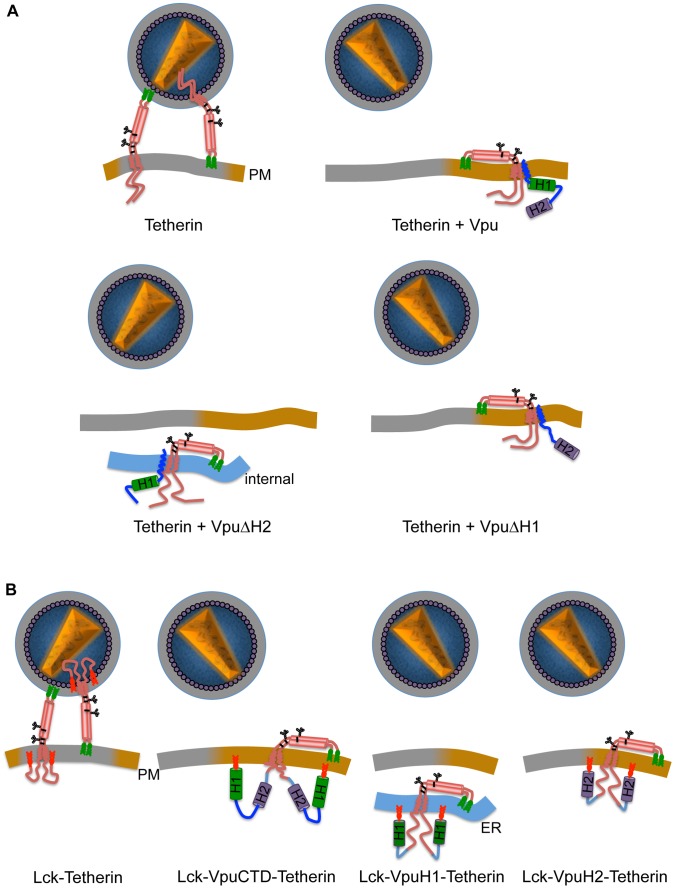
Models depicting the effect of Vpu on Tetherin. (**A**) Model of how Vpu acts to remove Tetherin from site of virion assembly: In the absence of Vpu, Tetherin is able to tether the virions at the cell surface by the partitioning of membrane anchors between cell and virion membranes. In the presence of Vpu, Tetherin and Vpu interact via their TMDs and Vpu removes Tetherin from sites of viral assembly, gray portion of the membrane. Vpu CTD H1, acts to cause intracellular retention while H2 displaces Tetherin from sites of virion assembly at the cell surface. (**B**) Recapitulation of these effects by fusing Tetherin CTD elements to the Tetherin protein.

Overall the data in this manuscript lead to a model for Vpu function (Figure 14) in which the TMD domain is responsible for directly binding Vpu to Tetherin, while H1 and the phosphorylated serine residues in the CTD are largely responsible for intracellular retention and degradation, and H2 is primarily responsible for displacing Tetherin from virions at the cell surface. Previous studies have demonstrated that the TMD of Tetherin determines its sensitivity to Vpu [37], [38], [40], [42], [44] and that residues in the Vpu TMD are important determinants of its activity [43], [44]. Moreover, Vpu and Tetherin can be coimmunoprecipitated and give a positive signal in bimolecular fluorescence complementation assays [34], [40]–[44], suggesting that they are components of the same protein complex, and NMR studies indicate that residues in the Tetherin TMD undergo chemical shifts in the presence of a fragment of the Vpu TMD in artificial lipid bilayers [44]. However, no previous study had unequivocally determined whether intact Vpu and Tetherin proteins directly bind to each other in the membranes of living cells.

We employed cysteine-scanning mutagenesis coupled with oxidation-induced cross-linking [45], which enabled us to demonstrate that Vpu and Tetherin interact directly in cell membranes. Additionally, we were able to identify several Tetherin and Vpu residues at the periphery of their respective TMDs that are in close proximity to each other. Previously reports have identified residues within the central portion of the TMD of Vpu that are important for Vpu function, when mutated in combination, and appear to interact with Tetherin [43], [44], While we did not observe cross-linking at these positions, nor did we observed functional consequences when these residues were mutated to cysteine, it is possible that the cell membrane inhibits penetration of oxidation reagent required for disulfide bond formation [51]. Thus, while our analysis identifies several points of contact between Tetherin and Vpu, it may underestimate the true extent of the interactions between the Tetherin and Vpu TMDs.

Our conclusion that there are multiple points of contact between Tetherin and Vpu TMDs is consistent with previous functional/genetic analyses. For example, mutation of individual residues that exhibit evidence of positive selection in the TMD of human Tetherin to rhesus macaque Tetherin counterparts, resulted in proteins that were at least partially sensitive to Vpu, whereas combinations of mutations gave fully Vpu resistant proteins, as summarized in Table 1 [37], [38]. Indeed, a variety of published mutational analyses of Tetherin [37], [38], [40], [42], [44] and Vpu [43], [44] have revealed that either large or multiple changes are required to completely disrupt the functional interaction between Vpu and Tetherin, suggesting multiple contact points exist between these two proteins, perhaps across almost the entire length of the TMDs (Table 1). Moreover, the finding that crosslinked residues in the Tetherin TMD appeared to occur on opposing sides of the TMD helix (Figure S1) suggests the possibility that there more an one binding site for Vpu on Tethe, perhaps in the context of a tetherin dimer.

Although there is significant concordance between the results of our crosslinking analysis and prior functional genetic analyses (Table 1), the above considerations likely underlie some of the discrepancies. Our analyses identified Tetherin TMD residues L22, L23, L24, G27, L29, L41, I43, F44, T45, and I46, as points of proximity to Vpu, and Vpu residues Q2, P3, I4, A7, V20, V25, I26, I27 as points of proximity to Tetherin, as summarized in Figures 3 and S1 and Table 1. Previous studies identified Tetherin TMD residues (L23, L24, G27, G28, V30, I33, I34, I36, L37, P40, L41, T45) as determinants of Vpu sensitivity and Vpu TMD residues (A10, A14, I15, A18, and W22) as key determinants of Tetherin antagonist activity (summarized in Table 1). Additionally, Tetherin residues (L23, L24, I26, G27, I28, V30, I34, I37, and L41) and Vpu residues (A10, A14, A18, and W22) have been implicated as potential points of interaction using coimmunoprecipitation or bimolecular fluorescence complementation assays [40], [42]–[44].

Notable discrepancies between our crosslinking data and prior functional/genetic assays include the observation that no cross-linking involving Tetherin V30 was detected, yet this residue appears to be a determinant of Vpu sensitivity and is involved in a NMR chemical shift [37], [38], [44]. We did however detect crosslink involving a neighboring Tetherin residue (L29) suggesting the possibility that perturbations of Tetherin TMD structure induced by V30 mutation might underlie its effect on Vpu sensitivity. Similarly, Vpu W22, mutation of which impairs Vpu function [43], [44], did not appear to directly contact Tetherin, but neighboring residues (V20 and V21) did appear to be in close proximity to Tetherin. Again these data suggest that residues which, when mutated, result in loss of function, may be involved in maintaining the overall structural conformation of TMDs rather than constituting points of interaction. Four residues within Vpu that formed the strongest crosslinks with Tetherin contributed to Vpu function, as the VpuQuad mutant was somewhat attenuated in its ability to antagonize Tetherin. However, these were not the only residues that were found to be in close proximity to Tetherin, and our findings underscore the notion that Vpu and Tetherin interact at multiple points within their respective TMDs as well as the importance of information obtained using different assays in interpreting how proteins interact, and the contribution of each component of the interaction to overall protein activity.

Clearly, TMD interactions recruit Vpu and Tetherin to each other, and thereby enable antagonism to occur through activities elicited by the Vpu CTD. Our functional dissection of the Vpu CTD suggested that Vpu possesses a degradation and downregulation-independent mechanism of antagonizing Tetherin. This explains the ability of mutated Vpu proteins to partially abrogate Tetherin antiviral activity in the absence of downregulation or degradation. Specifically, we found that Vpu can cause displacement of Tetherin from assembling virions at the cell surface.

Analysis of individual domains within the CTD of Vpu, in the context of mutant Vpu proteins, or when artificially ‘recruited’ to Tetherin in the context of Lck-CTD-Tetherin fusion proteins, revealed that either H1 or H2 domains of the CTD could elicit anti-Tetherin activity. Our analysis complements previous data that determined that an ExxxLV motif within H2 plays a role in directing Tetherin localization [50]. However our data further suggests that the Vpu H2 and the ExxxLV motif within it can act at the PM to displace Tetherin from sites of viral assembly. Precisely what role the ExxxLV motif plays in this process is unclear. It is possible that it is required for the structural integrity of H2, or forms part of a cofactor binding site. The fact that ExxxLV is required both for displacement of Tetherin from particle assembly sites at the cell surface, as well as determining the intracellular fate of Tetherin [50] suggests that its integrity is generally required for Vpu functions. Conceivably, displacement from assembly sites could be a first step in an H2-dependent multi-step process that leads to the downregulation and degradation of Tetherin. Further analyses revealed that the Lck-H1-Tetherin fusion protein was predominantly localized to the ER. This finding is consistent with previous reports that a Vpu mutant lacking H2 localized to new internal compartments [26] and suggests that Vpu H1 has a discrete ER retention or retrograde trafficking signal, which may play a role in antagonizing Tetherin [56].

Ultimately, in order to be efficiently released by infected cells, assembling virions require Tetherin to be absent from sites of viral assembly. Previous reports have revealed how Vpu causes Tetherin sequestration to internal compartments and downregulation from the cell surface, as well as decreased Tetherin stability [26]–[31], [33]–[36], [41], [49]. Here we have shown that Vpu can also displace Tetherin from sites of virus assembly without removing it from the cell surface, employing an activity that appears largely contained within the H2 domain of the Vpu CTD. Thus, it appears that Vpu can mobilize multiple mechanisms to abrogate the restriction imposed on HIV-1 by Tetherin.

## Materials and Methods

### Plasmid construction

Codon optimized Vpu [57] was mutated to Vpu^2/6^ (S52A S56A) using overlap-extension PCR and then inserted into the expression vector pCR3.1 (Invitrogen) with the sequence EcoRI-CoVpu-XhoI-FLAG(MDYKDHG-stop)-NotI. This Vpu^2/6^-FLAG expression vector was used as the parental plasmid to mutate each individual TMD amino acid residue to a cysteine residue using mutagenic 5′ PCR primers, or overlap-extension PCR.

Construction of plasmids expressing Tetherin-HA and TetherinΔGI-T45I-HA proteins were described previously. Briefly, an HA sequence was inserted at nucleotide position 463 in the Tetherin cDNA. Thereafter, G25 and I26 were deleted and a T45I mutation introduced [38]. WT and T45I-mutant Tetherin cDNAs were then mutated, to generate the parental backbones Tetherin^CC/SS^, harboring C9S and C20S mutations, which were then used to generate the individual cysteine point mutants in the Tetherin TMD. All mutagenesis was accomplished by using overlap-extension PCR.

Plasmids expressing Lck-Tetherin-HA, Lck-VpuCTD^2/6^-Tetherin-HA, Lck-VpuH1-Tetherin-HA, Lck-VpuH2-Tetherin-HA chimeras were generated in the context of a Tetherin-HA expression plasmid using overlap-extension PCR [58]. The cDNAs encoding Tetherins without their GPI anchor (ΔGPI) were generated by inserting a stop codon after the inserted HA epitope. The VpuCTD^2/6^, H1 and H2 sequences were defined, respectively, as residues E28-L81, I32-A49, and E57-L81 of NL4-3 Vpu. The Lck-Tetherin constructs were inserted into the retroviral vector pLHCX (Clontech) to generate stable cell lines expressing these proteins.

The proviral plasmids pNL4-3(WT), Vpu deficient pNL4-3(ΔVpu) and pNL4-3(Vpu^2/6^) (S52A, S56A) were previously described or were generated by overlap-extension PCR [59]. The proviral plasmid pNL4-3 (MA-YFP) that contained a YFP cDNA inserted into the stalk region of MA was previously described [60]. The various Vpu mutants, VpuDelR34-E47 (VpuΔH1), VpuE59Stop (VpuΔH2), and Vpu^2/6^ΔH1 were generated using overlap-extension PCR and inserted into pNL4-3 and pNL4-3 MA-YFP via EcoRI/NheI digestion, and into the HIV-1 based vector, V1B-GFP, via MfeI/KpnI digestion and ligation. For some Vpu mutations (S56A, E59Stop, E59A, L63A, V64A) changes also introduced mutations into the extreme N-terminus of the overlapping Env ORF. However, these were all at positions that were intrinsically variable in natural HIV-1 sequence, and where possible the Env changes were conservative. None of the Env mutations affected the yield of infectious HIV-1 particels in the absence of Tetherin. All constructs were sequence verified. Oligonucleotide sequences are available upon request.

### Cell line construction and maintenance

The cells HEK293T and HeLa-TZM cells expressing CD4/CCR5 and a LacZ reporter gene under control of the HIV-1 LTR were maintained in DMEM media containing 10% fetal calf serum and gentamycin (2 µg/ml, Gibco). HEK293T cells were transduced with pLHCX (Clontech) based retroviral vectors expressing genes of interest and selected with hygromycin (50 µg/ml) (MediaTech, Inc) to generate cell lines expressing Tetherin-HA, [38], Lck-Tetherin-HA, Lck-VpuCTD^2/6^-Tetherin-HA, Lck-VpuH1-Tetherin-HA, Lck-VpuH2-Tetherin-HA, Lck-TetherinΔGPI-HA, Lck-H1-TetherinΔGPI-HA, and Lck-H2-TetherinΔGPI-HA.

### Cysteine cross-linking

Oxidation induced cytsteine cross-linking was executed using a protocol adapted from [45]. Specifically, HEK293T cells were seeded at 2.5×10^5^ cells/well in 12 well plate and co-transfected the following day with 500 ng of a plasmid expressing a Vpu^2/6^-FLAG TMD cysteine mutant and 400 ng of a plasmid expressing a Tetherin^CC/SS^-HA TMD cysteine mutant. At 40 hours post transfection, the cells were incubated on ice and the media was replaced with oxidizing solutions. The oxidizing solutions were added to the cells by simultaneously adding 135 µl of each of two TMKP buffer solutions (10 mM Tris-HCl (pH 7.5), 15 mM MgCl_2_, 10 mM KCL, protease inhibitor cocktail (1 tablet/10 ml, Roche) containing either Copper sulfate (4 mM) or 1,10-Phenanthroline (12 mM, Sigma) to give a final concentrations of 2 mM and 6 mM, respectively. After mixing and incubation on ice for ten minutes, the cells were disrupted using a 550 Sonic Dismembrator probe sonicator (Fisher-Scientific) at a setting of 2 and incubated on ice for a further 10 mins. The lysates then received 40 µl each of additional TMKP Copper sulfate (6 mM) and Phenanthroline (18 mM) for a final concentration of 2.2 and 6.6 mM, respectively. The reaction was mixed and incubated for 10 mins on ice and then stopped by the addition of 20 mM N-ethylmaleimide and 5 mM EDTA. Lastly, 4× Nu-PAGE (Invitrogen) sample buffer was added (without reducing agent) and the samples were analyzed by SDS-PAGE and Western blotting. For spontaneous crosslinking assays in the absence of oxidizing agent, 200 ng of a plasmid expressing a Vpu^2/6^-FLAG TMD cysteine mutant and 200 ng of Teth^CC/SS^-I46C-HA DNA, and 50 or 100 ng Tetherin^CC/SS^ DNA were used to transfect HEK293T cells. At 48 hrs. post-transfection, the cells were harvested with NuPAGE sample buffer without reducing agent.

### Immunoprecipitation of Tetherin-Vpu complexes

For immunoprecipitation of Vpu-Tetherin cross-linked complexes, HEK293T cells were cotransfected and treated as above with oxidizing agents. Immunoprecipitation conditions were as described previously [61]. After oxidation, cells were lysed in detergent-rich denaturing buffer including protease inhibitor tablets (Roche) and 5 mM N-ethylmaleimide to inhibit further cross-linking. After centrifugation to clear cell debris, cell lysates were diluted 5-fold with the same buffer, except SDS was substituted with NP-40 (to a final concentration of 1%), in order to dilute SDS to 0.1% prior to immunoprecipitation. The cleared lysates were incubated with anti-HA.11 monoclonal antibody (Covance), and complexes were isolated with Protein-G Sepharose 4 Fast Flow (GE Healthcare). Immunoprecipitates and unfractionated cell lysates were analyzed by Western blotting. For non-denaturing immunoprecipitation of non-cross-linked Tetherin-Vpu complexes procedures were as described previously [34].

### Virion yield assays

HEK293T cells were seeded in 24 well plates at a concentration of 1.5×10^5^ cells/well and transfected the following day using polyethylenimine (PEI) (PolySciences) with 500 ng of pNL4-3(WT) or pNL4-3(ΔVpu) along with various amount of a Tetherin expression plasmid (25 ng to 200 ng), with the empty pCR3.1 vector used as a filler. Following transfection, media was replaced with fresh DMEM (1 ml) and supernatants were collected and filtered through a 0.2 µm PVDF filter (Millipore) at 40 hours post transfection. Infectious virion yield was determined by inoculating HeLa-TZM indicator cells seeded in a 96 well plate at 1.5×10^4^ cells/well with 10 µl of serially diluted virus. At 48 hrs. post-infection, beta-galactosidase activity was determined using GalactoStar reagent following manufacturer's instructions (Applied Biosystems). Physical particle yield was determined by layering 750 µl of the virion containing supernatant onto 800 µl of 20% sucrose in PBS and centrifugation at 20,000×g for 90 minutes at 4°C. Virion pellets were then analyzed by Western blotting.

### Effects of Vpu mutants on Tetherin expression

HEK293T cells seeded at 4×10^6^ cells/10 cm plate were cotransfected with VSV-G (1 µg), pCRV1/HIV-1GagPol (5 µg), and a V1B-GFP based HIV-1 vector expressing a WT or mutant Vpu protein DNA (5 µg). At 40 hours post-transfection, the supernatants were filtered (0.2 µm PVDF, Millipore). The virus titers were determined by measuring the fraction of GFP positive cells after infection of HEK293T cells with serially diluted virus. Thereafter, HEK293T cells stably expressing Tetherin-HA were seeded at 1.5×10^5^ cells/well in a 24 well plate. The following day, cells were transduced at a MOI of 0.3 with the aforementioned V1B-GFP vectors expressing various Vpu proteins. After 48 hrs, the cells were harvested with 5 mM EDTA in PBS and blocked with 3% rabbit serum in PBS at 12°C and incubated with rabbit anti-HA conjugated to SureLight APC 1∶50 (Colombia Biosciences) or anti-huTetherin antibody conjugated to APC (Biolegend) and DAPI (Invitrogen). FACS analysis using a FACS (BD LSR II) was used to determine the relative amount of surface Tetherin expression in GFP-positive and DAPI-negative cells. Alternatively, cells were infected with the same panel of viruses at an MOI of 3, lysed 40 hours later with NuPage sample buffer, and Tetherin expression analyzed by Western blotting.

### Western blot assays

Pelleted virions, cell lysates and immunoprecipitates were resuspended in SDS-PAGE loading buffer and separated on NuPAGE Novex 4–12% Bis-Tris Mini Gels (Invitrogen) or on BioRad 12% Tris-HCl gels with Laemmli sample buffer. Proteins were blotted onto nitrocellulose membranes (HyBond, GE-Healthcare). The blots were then probed with mouse anti-HIV-1 capsid (NIH), rabbit anti-HA (Rockland), mouse anti-FLAG (Sigma), mouse anti-GFP (Roche) or rabbit anti-tubulin (Santa-Cruz Biotechnology) primary antibodies. Bound primary antibodies were detected using HRP-conjugated secondary antibodies (Jackson Laboratories) and chemiluminescent detection reagents (Pierce) or with fluorescently labeled secondary antibodies, and a LI-COR Odyssey scanner (LI-COR biosciences).

### Microscopy

To determine the subcellular distribution of Tetherin mutants and chimeras, HEK293T cell lines stably expressing HA-tagged Tetherin, Lck-Tetherin, Lck-VpuCTD^2/6^-Tetherin, Lck-VpuH1-Tetherin, or Lck-VpuH2-Tetherin were seeded at 1×10^5^ cells on 3.5-cm glass bottomed dishes coated with poly-L-lysine (Mattek). Two days later the cells were fixed with 4% paraformaldehyde (PFA), washed with 5 mM glycine in PBS followed by 0.5% TritonX-100 to permeabilize the cells. Cells were blocked with 1% bovine serum albumin in PBS and incubated with anti-HA.11 monoclonal antibody (Covance) followed by goat antimouse IgG conjugated to Alexafluor-594 (Molecular Probes). Cells were counterstained with DAPI (Invitrogen).

To assess the colocalization of Tetherin with virus particles, cell lines expressing Tetherin mutants or chimeras were seeded at 5×10^4^ cells per/dish. At 48 hrs later, the cells were cotransfected, using PEI, with pNL4-3(WT), pNL4-3(ΔVpu). pNL4-3(Vpu^2/6^) pNL4.3 (VpuΔH1), pNL4.3(Vpu^2/6^ΔH1), pNL4.3(VpuΔH2), pNL4.3(Vpu^2/6^ΔH2), or pNL4.3(Vpu^2/6^H2A3), mixed with a matching counterpart expressing MA-YFP at a ratio of 1∶1 (200 ng each). At 48 hours post-transfection, cells were processed as described above, but were not permeabilized. Imaging was performed as described previously [62], but in brief, a z-series of images were taken to capture the top of the cell. Several regions of each dorsal cell surface were selected to measure colocalization between Tetherin and viral particles and the mean Pearson's correlation coefficient calculated using DeltaVision software, as previously described [62].

### Tetherin incorporation into virions

To assess the incorporation of Tetherin into virions a protocol was adapted from previous work [14]. HEK293T cell lines stably expressing Lck-Tetherin, Lck-TetherinΔGPI, Lck-H2-TetherinΔGPI, or Lck-H1-TetherinΔGPI were seeded at a concentration of 5×10^5^ cells/well in 6 well plates and infected the next day by VSV-G pseudotyped NL4-3 HIV-1(Vpu^2/6^) ΔNef::GFP or NL4-3 HIV-1(ΔVpu) ΔNef::GFP virus at an MOI of 1. The next day the media was replaced with 4 mls of fresh media. The supernatants were harvested the following day, filtered (0.2 µm PVDF, Millipore), layered onto 8 mls of 20% sucrose in PBS and centrifuged at 90,000×g for 90 minutes. The isolated viral pellets were suspended in NuPAGE sample loading buffer and the whole cell lysates were analyzed by Western blotting.

## Supporting Information

Figure S1 **A stereoscopic view of the Tetherin TMD residues that formed crosslinks.** A stereoscopic model of the Tetherin TMD, residues 22–45, in which the positions of α-carbons from NMR structures from are modeled using PyMol. The shades of green represent the varying degrees to which crosslinking to Vpu occurred. Red represents no observable crosslinking, see inset for key.
(TIF)

Figure S2 **Vpu CTD H1 reduces cell surface Tetherin expression and induces relocalization to the ER in context of the Lck-H1-Tetherin protein.** (**A**) 293T cells were cotransfected with a plasmid encoding GFP and a plasmid encoding various Tetherin proteins. Cells were then stained with an antibody against Tetherin that was directly conjugated to APC. Living, singlet cells expressing GFP were gated and the level of Tetherin localized at the cell surface that was reported as the median fluorescence intensity. (**B**) 293T cells stably expressing Lck-H1-Tetherin (top row of panels) or Lck-Tetherin (bottom row of panels) were transiently transfected with a plasmid expressing the ER marker DsRED-ER (red). Fixed and permeabilized cells were stained with α-HA to reveal the Lck-Tetherin-HA proteins (Alexa-488, green). Merged image show in the right column and scale bar is 5 µm.
(TIF)

Figure S3 **Relative cell surface expression of Tetherin containing proteins without a GPI anchor.** Single cell clones of 293T cells stably expressing the indicated proteins Lck-TetherinΔGPI-HA, Lck-H1-TetherinΔGPI-HA, or Lck-H2-TetherinΔGPI-HA were cell surface stained using an antibody against HA directly conjugated to the flourochrome APC. Living and singlet cells were then gated and the Tetherin signal iss reported as the median fluorescent intensity.
(TIF)
