# Supplementary figures and images for: Vpu Binds Directly to Tetherin and Displaces It from Nascent Virions
Source: PLoS Pathog. 2013 Apr 25;9(4):e1003299. doi: 10.1371/journal.ppat.1003299 (PMC3635990; doi:10.1371/journal.ppat.1003299)

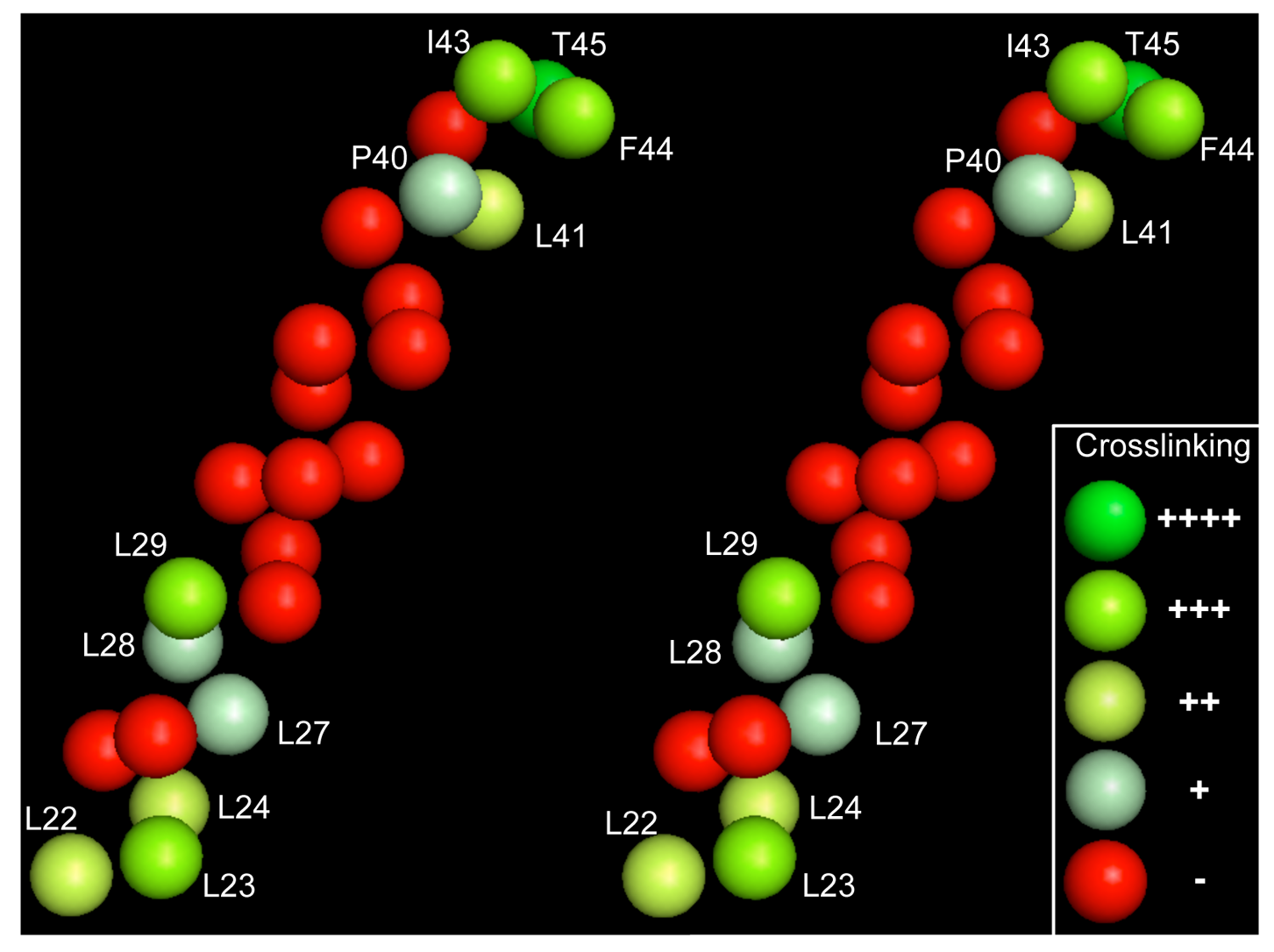

Supplement: Figure S1 — A stereoscopic view of the Tetherin TMD residues that formed crosslinks. A stereoscopic model of the Tetherin TMD, residues 22–45, in which the positions of α-carbons from NMR structures from are modeled using PyMol. The shades of green represent the varying degrees to which crosslinking to Vpu occurred. Red represents no observable crosslinking, see inset for key. (TIF) [file ppat.1003299.s001.tif]

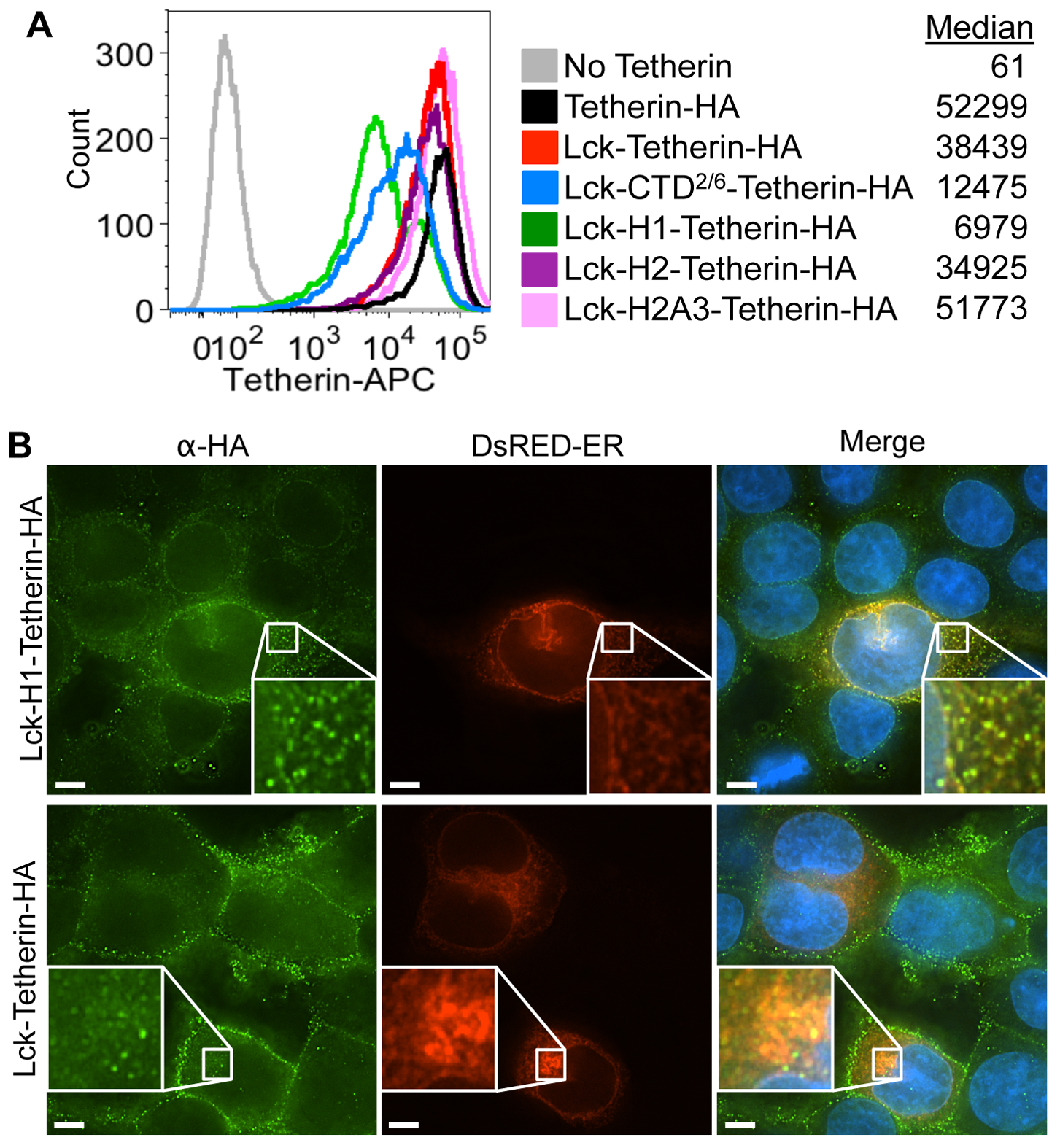

Supplement: Figure S2 — Vpu CTD H1 reduces cell surface Tetherin expression and induces relocalization to the ER in context of the Lck-H1-Tetherin protein. (A) 293T cells were cotransfected with a plasmid encoding GFP and a plasmid encoding various Tetherin proteins. Cells were then stained with an antibody against Tetherin that was directly conjugated to APC. Living, singlet cells expressing GFP were gated and the level of Tetherin localized at the cell surface that was reported as the median fluorescence intensity. (B) 293T cells stably expressing Lck-H1-Tetherin (top row of panels) or Lck-Tetherin (bottom row of panels) were transiently transfected with a plasmid expressing the ER marker DsRED-ER (red). Fixed and permeabilized cells were stained with α-HA to reveal the Lck-Tetherin-HA proteins (Alexa-488, green). Merged image show in the right column and scale bar is 5 µm. (TIF) [file ppat.1003299.s002.tif]

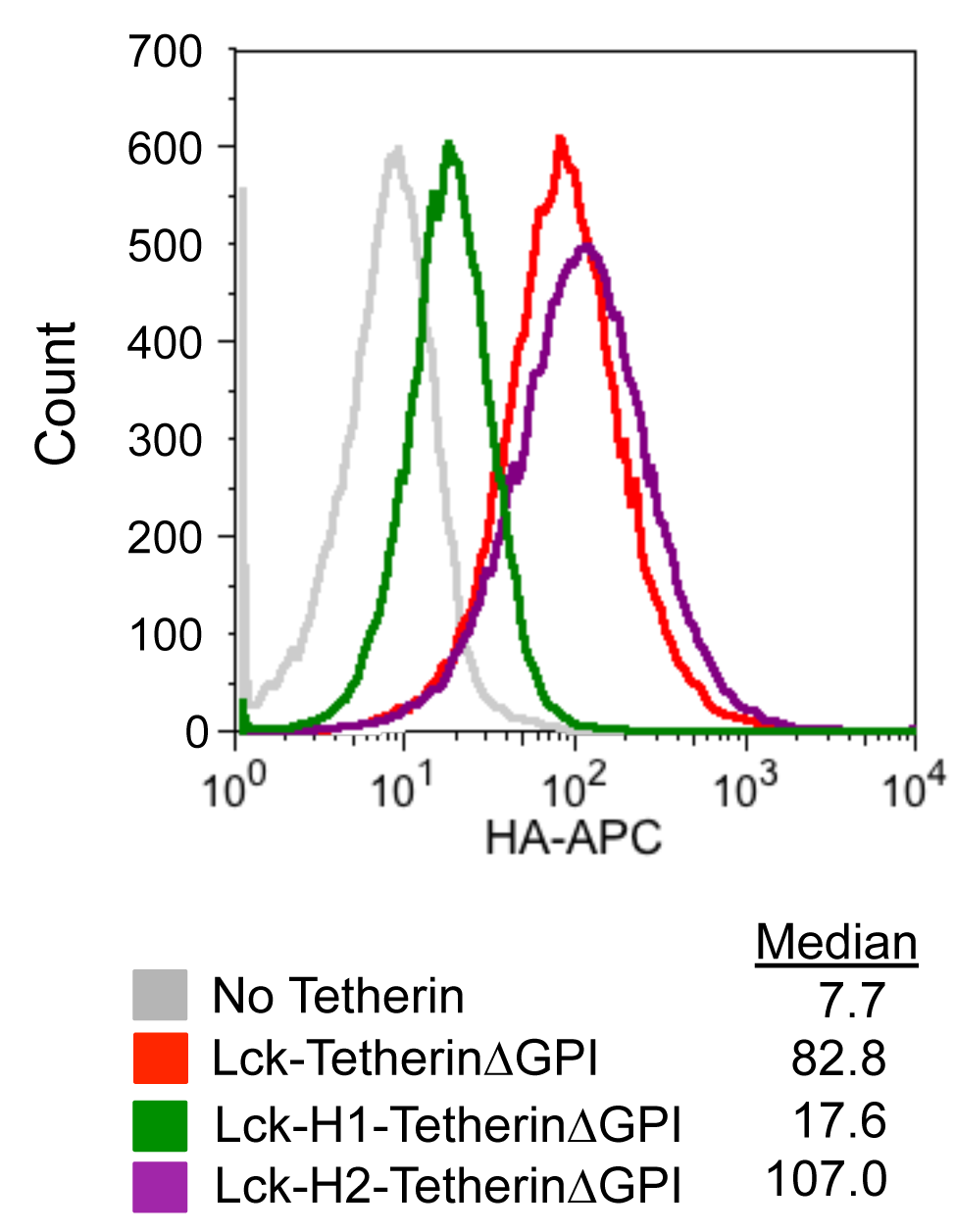

Supplement: Figure S3 — Relative cell surface expression of Tetherin containing proteins without a GPI anchor. Single cell clones of 293T cells stably expressing the indicated proteins Lck-TetherinΔGPI-HA, Lck-H1-TetherinΔGPI-HA, or Lck-H2-TetherinΔGPI-HA were cell surface stained using an antibody against HA directly conjugated to the flourochrome APC. Living and singlet cells were then gated and the Tetherin signal iss reported as the median fluorescent intensity. (TIF) [file ppat.1003299.s003.tif]
